# Angular spectrum-encoded single-shot ultrafast photography

**DOI:** 10.1038/s41377-026-02289-3

**Published:** 2026-06-05

**Authors:** Chen Huang, Chunqi Jin, Yi Chen, Xin Zhang, Chunrui Wang, Hanyu Zheng, Xueqing Liu, Qidai Chen, Junjie Sun, Fei Chen

**Affiliations:** 1 Jilin Provincial Key Laboratory of High Power Laser Technology and Application, Changchun Institute of Optics, Fine Mechanics and Physics, Chinese Academy of Sciences, Changchun, 130033 China; 2 University of Chinese Academy of Sciences, Beijing, 100049 China; 3 State Key Laboratory of Integrated Optoelectronics, College of Electronic Science and Engineering, Jilin University, Changchun, 130012 China

**Keywords:** Imaging and sensing, Near-infrared spectroscopy

## Abstract

Capturing transient events on ultrafast time scales demands imaging at up to trillions of frames per second (Tfps). Yet leading methods, including compressed sensing-based photography, time-resolved shadowgraphy, and other active approaches, are limited by bulky optics, high cost, and repeated measurements. Here, we propose angular spectrum-encoded single-shot ultrafast photography (ASUP). ASUP combines the time-wavelength mapping of a chirped-pulse probe with dispersion-encoded angular spectrum information. The latter is realized by a multilayer dielectric thin-film photonic chip, inversely designed using a deep Q-network (DQN) reinforcement-learning framework. This chip performs pixel-level encoding without the need for bulky dispersive optics. An enhanced residual convolutional neural network with Transformer blocks then decodes the measurements to reconstruct high-fidelity ultrafast dynamics. In experiments, ASUP achieves 0.83 Tfps with six frames in a single exposure and captures picosecond laser-induced damage and plasma dynamics in metal films. ASUP delivers performance comparable to state-of-the-art ultrafast photography while remaining compact, highly integrated, and cost-effective. It overcomes key limitations of existing ultrafast imaging and offers a scalable solution for high-speed optical diagnostics, laser-matter interactions, and transient phenomena studies.

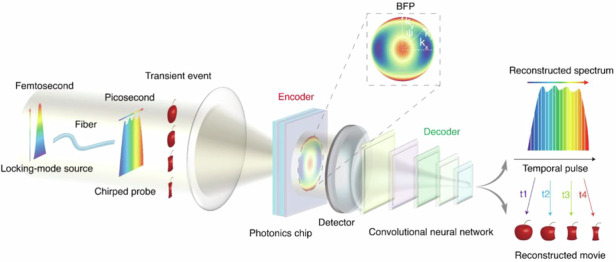

## Abstract (web summary)

A chip-scale angular spectrum encoding approach enables single-shot ultrafast photography and spectral imaging, offering compact, integrated high-speed optical measurement.

## Introduction

The exploration of extreme physical, chemical, and biological events has driven ultrafast imaging to the forefront of contemporary science. Numerous transient phenomena have been probed down to the atomic time scale^1,2^, including nonlinear effects^3^, photochemistry^4–6^, laser-induced plasmas^7,8^, fluorescence and incandescence^9,10^, microbial discharge dynamics^11^, and laser micro- and nano-processing^12^. Capturing these ultrafast events demands imaging speed on trillions of frames per second (Tfps). Yet, such frame rates far exceed the intrinsic temporal resolution of conventional silicon-based sensors (CCD or CMOS), whose physical response times are fundamentally limited^13^. To overcome the constraints of conventional sensors, a range of ultrafast imaging technologies has been developed, including burst cameras^14,15^, rotating-mirror cameras, in situ storage image sensors, and streak cameras, achieving frame rates from billions to trillions of frames per second (Gfps to Tfps). However, these techniques suffer from restricted fields of view, limited spatial resolution, bulky hardware, and high costs. The advent of ultrafast pulsed lasers has further enabled active ultrafast imaging schemes, such as time-stretch imaging^16,17^ and pump-probe methods^18,19^. While time-stretch imaging translates spatial information into temporal profiles for sequential frame acquisition, its frame rate is constrained by laser repetition frequency. Similarly, pump-probe imaging achieves femtosecond temporal resolution but requires repeated measurements, making it inherently unsuitable for non-repetitive or stochastic events such as explosive shockwaves or rogue waves.

In recent years, single-shot ultrafast photography has emerged as a transformative solution, capable of capturing entire sequences of transient dynamics within a single exposure^20^. Representative examples include compressed ultrafast photography (CUP)^21^, UV-CUP^22^, T-CUP^23^, and CUST^24^, which utilize encoded sparse sampling and compressive sensing to achieve Tfps imaging speeds. Yet, issues of reliance on specialized hardware continue to restrict their general applicability, underscoring the demand for compact and accessible alternatives. In parallel, active single-shot methods based on optical encoding strategies have achieved remarkable temporal resolution by leveraging spectral frame separation (STAMP^25^, SF-STAMP^26^, and LA-STAMP^27^); Fourier space filtering (FRAME^28^ and TSFM^29^); polarization encoding (PUMP^30^ and WPMSI^31^); time-space mapping based on phase matching (FINCOPA^32^); spatial-domain multiplexing (FTOP^33^); and angular encoding (FDT^34^ and LIFT^35^). Despite their successes, these approaches still require bulky dispersive optics, diffractive elements, microlens arrays, or echelon gratings, which hinder miniaturization and integration into compact photonic platforms.

**Fig. 1:**
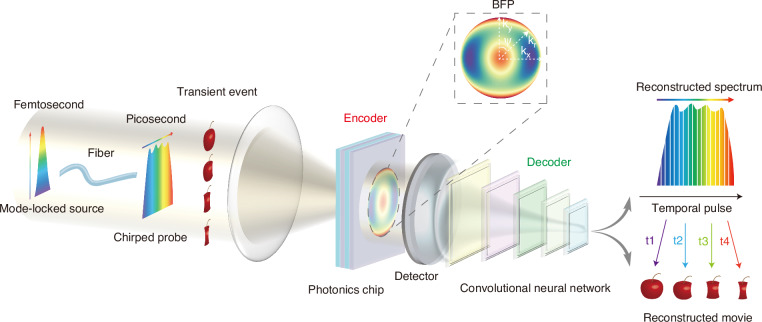
The concept of the ASUP. The mode-locked femtosecond laser is stretched into a picosecond laser by a chirped fiber Bragg grating, serving as a probe light for transient time measurement. Simultaneously, the laser’s spectrum is also temporally stretched as a chirped laser. The probe light is then directed through a lens to achieve incidence at various angles onto a photonic chip. After passing through the chip, the light converges onto a detector, forming the back focal plane (BFP) image, thereby encoding the incident spectrum. This encoded spectral information is subsequently decoded using a convolutional neural network (CNN), enabling the reconstructed spectrum and the ultrafast movie

These challenges underscore a pressing need for ultrafast imaging technologies that are simultaneously compact, scalable, cost-effective, and capable of single-shot capture at Tfps-class speeds. Addressing this demand, here we propose and demonstrate angular spectrum-encoded single-shot ultrafast photography (ASUP), a compact ultrafast imaging technique that realizes six-frame single-shot imaging at 0.83 Tfps, representing a significant advance toward chip-scale ultrafast diagnostics. As illustrated in Fig. 1, ASUP adopts an active detection approach, leveraging the intrinsic interactions between chirped pulse dispersion and temporal encoding to achieve multi-frame transient imaging. Using a photonic chip consisting of a multilayer dielectric film, different frequency (wavelength) components of the detected light can be extracted. It is worth noting that the multilayer structure is optimally designed via deep Q-network (DQN) reinforcement learning (RL) to achieve the most effective stacking configuration. This chip exhibits an angle-dependent spectral response, encoding the angular spectrum of the probe light at the pixel level using a single thin-film structure. Given the complex spatiotemporal mapping involved, we employ an enhanced residual convolutional neural network with Transformer blocks (ERCNN) to decode the collected probe light and reconstruct the original spectral information, enabling the high-fidelity retrieval of ultrafast dynamic events. By removing dependence on streak cameras and bulky dispersive optics, ASUP offers a fundamentally new paradigm for real-time ultrafast diagnostics, with potential applications across physics, materials science, and biomedical imaging.

## Results

### Design principles and simulation results

Different wavelengths λ within an ultrashort mode-locked pulse propagate at varying velocities when passing through a dispersive medium, causing gradual temporal stretching of the pulse. When the total group delay dispersion $D$ significantly exceeds the square of the initial pulse duration ${t}_{0}^{2}$, the mapping relation between time t and frequency $w$ ($w\propto 1/\lambda$) can be expressed as $t={Dw}$ ^36,37^. Under this condition, known as the temporal Fraunhofer regime, the input optical spectrum is directly mapped onto the output temporal wavefront as^38^

1 $${u}_{{out}}\left(t\right)={h}_{T}\exp \left(-i\frac{{t}^{2}}{2D}\right)\int {u}_{{in}}({t}^{{\prime} })\exp \left(-i\frac{{t}^{{\prime} }t}{D}\right)d{t}^{{\prime} }$$

By stretching pulses from a mode-locked laser, discrete temporal distributions for different frequencies (wavelengths) can be obtained. To selectively extract probe signals of varying wavelengths, we propose using a photonic chip featuring angular dispersion characteristics in Fourier space. The angular spectrum $A({k}_{x},{k}_{y})$ represents a decomposition of the in-plane electric field ${E}_{{in}}(x,y)$ into a continuum of plane waves, each characterized by a transverse wave vector (${k}_{x},{k}_{y}$). The components ${k}_{x}$ and ${k}_{y}$ depend primarily on the incident angle $\theta$, defined as ${k}_{{x\backslash y}}={k}_{0}\sin \theta$. It describes how much of the field propagates in a given direction in Fourier space. This spectrum is obtained by a 2D Fourier transform:

2 $$A\left({k}_{x},{k}_{y}\right)={\mathscr{F}}\left[{E}_{{in}}\left(x,y\right)\right]=\iint {E}_{{in}}\left(x,y\right)\exp [-i({k}_{x}x+{k}_{y}y){dxdy}$$

Each plane wave component in the angular spectrum propagates at an angle determined $\theta$ and contributes to the total field as:

3 $${E}_{{in}}\left(x,y\right)=\iint A\left({k}_{x},{k}_{y}\right)\exp [i({k}_{x}x+{k}_{y}y)d{k}_{x}d{k}_{y}$$

When this field passes through the photonic chip, the chip imposes a spatial filtering effect described by the optical transfer function $t({k}_{x},{k}_{y})$, which selectively transmits or suppresses certain angular components. The output field becomes:

4 $${E}_{{out}}\left(x,y\right)=\iint t({k}_{x},{k}_{y})A\left({k}_{x},{k}_{y}\right)\exp [i\left({k}_{x}x+{k}_{y}y\right)d{k}_{x}d{k}_{y}]$$

To highlight angular selectivity, it is useful to rewrite the integral in polar coordinates:

5 $${E}_{{out}}\left({k}_{r},\psi \right)=\iint t({k}_{r},\psi )A({k}_{r},\psi )\exp [i{k}_{r}{rcos}(\alpha -\psi )]{k}_{r}d{k}_{r}d\psi$$

Where ${k}_{r}=\sqrt{{k}_{x}^{2}+{k}_{y}^{2}}$ is the radial wavevector. Here, $\alpha =\arctan (x/y)$ is the angle in real space between the radial direction r and the y-axis, and $\psi =\arctan ({k}_{x}/{k}_{y})$ is the angle in Fourier space between ${k}_{r}$ and ${k}_{y}$. The optical transfer function $t({k}_{r},\psi )$ in p polarization is described as: (see detailed derivation in Supplementary Information Note 1)

6 $$t\left({k}_{r},\psi \right)=\sin \varphi \cos \psi \left({tp}({k}_{x},{k}_{y})-{ts}({k}_{x},{k}_{y})\right)+{\sin }^{2}\psi {ts}({k}_{x},{k}_{y})+{\cos }^{2}\psi {tp}({k}_{x},{k}_{y})$$

Where ${t}_{s}$ and ${t}_{p}$ are the transmission amplitudes of s polarization and p polarization, respectively. It emphasizes how angular filtering in $\left({k}_{r},\psi \right)$ space leads to controlled field shaping in real space. By following this concept, we design an angle-selective multilayer photonic chip (MOC) based on multilayer dielectric films, enabling distinct angular spectrum intensity profiles across different wavelengths.

To achieve optimal angle-dependent properties in the MOC, we employ a DQN approach for inverse design optimization of the multilayer film structure parameters. Iterative optimization algorithms such as ant colony optimization, genetic algorithms, and adjoint methods have previously shown success in inverse design of photonic components^39–41^. However, breakthroughs in fabrication and materials science have substantially increased the number of accessible design parameters, causing an exponential growth in the parameter search space. This complexity often leads optimization processes into local minima (a problem mathematically defined as non-convexity)^42,43^. The RL-based DQN method is particularly adept at addressing such challenges and has demonstrated remarkable efficiency in multilayer film design tasks.

**Fig. 2:**
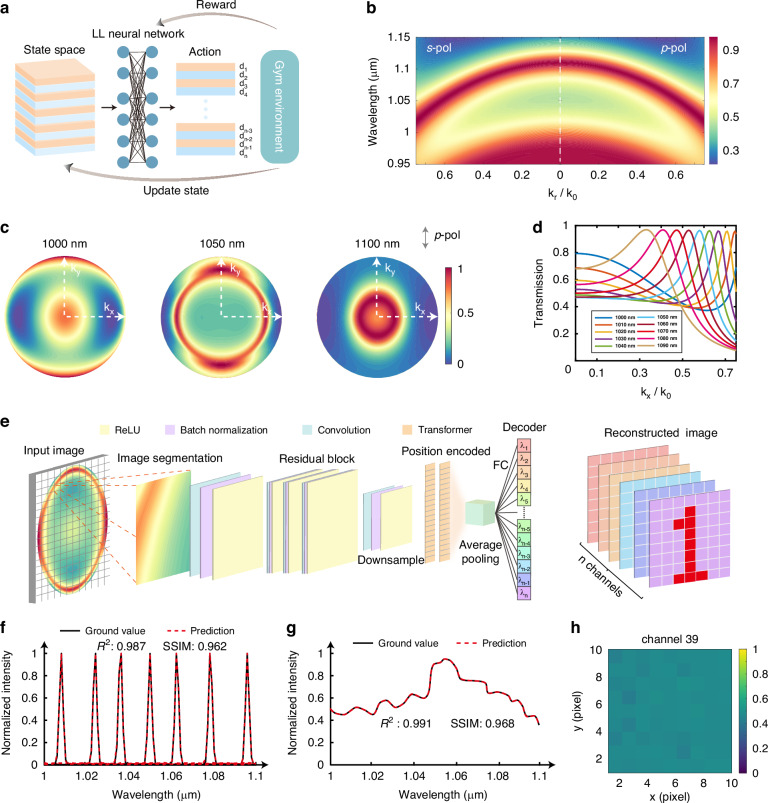
Design of the photonic chip and intelligent image reconstruction. **a** Workflow of inverse design for the MOC based on a DQN, where LL neural network denotes a two-layer linear neural network. **b** Simulated transmission coefficients of the MOC as functions of wavelength and wavevector for s- and p-polarized light. **c** Simulated BFP images of the MOC under three different incident wavelengths: 1000 nm, 1050 nm, and 1100 nm. Gray arrows indicate the polarization direction of the incident light. **d** Transmission coefficient function at different wavelengths. **e** Workflow of image reconstruction using the ERCNN model, where the input is the BFP image of the MOC and the output is a reconstructed image with 101 spectral channels. **f**, **g** Spectral curves predicted by the ERCNN model (red dashed lines) compared to the ground value of spectral curves. **h** Pixel-wise encoded reconstructed image at the 39th spectral channel

In the proposed DQN framework, the multilayer film’s state (material arrangement and thicknesses) is represented as state space S, fed into the RL model to obtain a reward value R. Different decision strategies are then selected according to the reward-derived action-value function Q. We construct a deep neural network to establish a nonlinear mapping between the S and Q. The inverse design workflow is illustrated in Fig. 2a. The TMM-Fast simulation environment^44^ interacts with the DQN agent in the Gym environment, and a replay buffer stores tuples in the form $({S}_{i},{A}_{i},{R}_{i},{S}_{i+1},{Terminal})$, capturing states, actions, and rewards during training. During training, two neural networks, the online network and the target network, predict the respective action-value functions Qo and Qt. The design of multilayer films is inherently a Markov decision process (MDP), and thus we employ the epsilon-greedy strategy to balance exploration and exploitation of the action space. Since DQN does not require initial configurations, the first 200 training iterations employ random control actions. The loss function minimized during training is defined as:

7 $${loss} = \frac{\left| Q_0 - \sum_{i=1}^{N} \left( R_i + \gamma Q_{t(\max)} \right) \right|}{N}$$

Where $\gamma$ is the discount factor, and ${Q}_{t(\max )}$ is the maximum value predicted by the target network. Through iterative updates, we achieve an optimal multilayer film structure that minimizes the loss. Detailed DQN design data and parameters are provided in Supplementary Information Note 2.

The transmittance of the MOC, calculated using the transfer matrix method (TMM)^45^, is illustrated in Fig. 2b, where left and right sections correspond to s- and p-polarizations, respectively. Using Eqs. (5) and (6), angle-dependent angular spectrum intensity maps (BFP images) are obtained at different wavelengths. Figure 2c demonstrates BFP images at wavelengths of 1000 nm, 1050 nm, and 1100 nm, respectively, with gray arrows indicating the p-polarization direction. Consequently, each pixel of the BFP image acquired by the MOC possesses a unique spectral encoding. Figure 2d illustrates the transmittance relationship between numerical aperture (NA) in the ${k}_{x}$ direction and ten distinct wavelengths, establishing a dispersive encoding suitable for angular spectrum information extraction.

We introduce an ERCNN model to decode the encoded angular spectrum information, integrating convolutional layers, residual blocks, and Transformer blocks. Before decoding, the BFP image is divided into smaller patches of 16 × 16 pixels, which serve as parallel inputs to the neural network. Figure 2e illustrates the complete deep learning-based reconstruction pipeline. The process begins with the ERCNN model performing initial feature extraction through a 3 × 3 convolution layer, followed by batch normalization and ReLU activation, expanding the feature channels from 1 to 64. This is followed by three pre-activation residual blocks that extract deeper local features. A subsequent 3 × 3 convolution with a stride of 2 downsamples the feature maps from 16 × 16 to 8 × 8 pixels. The resulting feature map is then reshaped into a sequence and enhanced with positional encoding, enabling a two-layer Transformer encoder to capture long-range dependencies and improve global feature representation. Global average pooling is then applied to produce fixed-length feature vectors, which are passed through fully connected layers to output the final n-dimensional spectral responses (ERCNN model details in Materials and methods).

The spectral responses targeted in this study range from 1000 nm to 1100 nm, discretized into 101 spectral channels. By independently training each segmented patch and reassembling the outputs, a reconstructed image with multiple spectral channels is obtained. Further implementation details are provided in Supplementary Information Note 3. Figure 2f demonstrates excellent spectral reconstruction accuracy, with SSIM of 0.962 and ${R}^{2}$ of 0.987 for spectra with 2 nm bandwidths. These results indicate that the proposed method can faithfully recover fine spectral features with high reliability. Random broadband spectra reconstructions (Fig. 2g) further validate robustness, showing SSIM of 0.968 and ${R}^{2}$ of 0.991. Figure 2h showcases a 39th channel reconstructed spectral image with a 2 nm bandwidth centered at 1038 nm, demonstrating the potential of our method to provide pixel-level spectral information, albeit with reduced spatial resolution (details in Supplementary Information Note 3). From the above simulation results, it can be seen that our method theoretically realizes an imaging spectrometer.

### Fabrication and characterization of the multilayer photonic chip

**Fig. 3:**
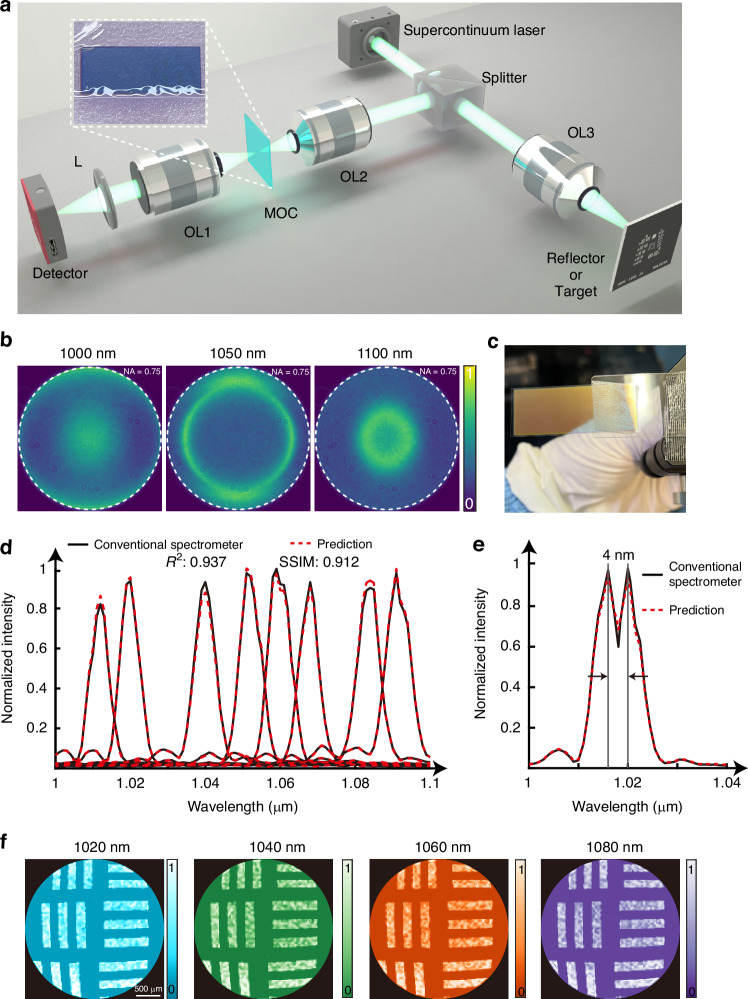
**Encoded angular spectrum and reconstructed image**. **a** Experimental setup for angular spectrum encoding. A supercontinuum laser source combined with an acoustic-optical tunable filter (AOTF) is used for wavelength selection. L denotes an achromatic lens serving as the imaging lens. The objective lenses (OL1 and OL2) enable the MOC to modulate the angular spectrum of the incident beam. The detector captures the modulated BFP image. OL3 functions as the imaging objective. The reflector and target can be interchanged depending on specific experimental requirements. **b** BFP images of the MOC at three different wavelengths: 1000 nm, 1050 nm, and 1100 nm. **c** Photograph of the fabricated MOC. **d** Comparison between reconstructed spectral data and ground-truth measurements at multiple central wavelengths. **e** Spectral resolution of the experimental device. In (**d**, **e**), solid lines represent measurements obtained using a commercial spectrometer, while dashed lines denote predictions generated by the neural network model. **f** Reconstructed spectral images at four representative wavelengths: 1020 nm, 1040 nm, 1060 nm, and 1080 nm

To experimentally validate this concept and quantitatively assess both the spectral reconstruction and imaging capabilities, we fabricated an MOC using ion beam deposition, with detailed structural parameters provided in Fig. S1c. A photograph of the fabricated MOC is shown in Fig. 3a, c. We constructed an imaging setup capable of acquiring both BFP images and spectrally resolved reconstructed images, as illustrated in Fig. 3a. This setup was used to characterize the angle-dependent transmission and spectral reconstruction performance of the MOC. A supercontinuum laser source coupled with an acoustic-optical tunable filter (AOTF), spanning a broadband range from 400 to 2400 nm, was employed to provide tunable monochromatic illumination. The laser beam was directed through a prism splitter and then into objective lens OL3 (0.5 NA). For BFP imaging, a reflector was placed after OL3 to reflect the light back through the same optical path—via the splitter, objective lens OL2 (0.75 NA), the MOC, and objective lens OL3 (0.75 NA)—before being focused by an achromatic cemented lens L onto the detector. Experimentally acquired BFP images at center wavelengths of 1000 nm, 1050 nm, and 1100 nm are shown in Fig. 3b, closely matching the simulated results in Fig. 2c. This agreement confirms that the MOC exhibits angle-dependent spectral dispersion and can encode angular spectrum in a wavelength-selective manner. To decode the angular spectrum-encoded information, we employed our custom-designed ERCNN model (workflow details are provided in Supplementary Information Note 4). Figure 3d displays the spectral reconstruction performance of our device across different wavelengths. The predicted spectra show strong agreement with measurements from a conventional commercial spectrometer, with SSIM of 0.912 and *R*^2^ of 0.937, indicating high reconstruction accuracy. These results experimentally validate the spectral sensing capability of our device. Furthermore, the spectral resolution of the device was determined to be 4 nm, as shown in Fig. 3e. To further verify pixel-level encoding and spectral image reconstruction, we replaced the reflector after OL3 with a resolution test target (USAF1951). Figure 3f presents the reconstructed spectral image of a selected region of the resolution target, revealing not only accurate spectral recovery but also good spatial resolution of approximately 100 µm. This spatial-spectral fidelity is critical for capturing transient phenomena in our ultrafast imaging applications.

### Ultrafast dynamics imaging with ASUP

Under ultrafast laser irradiation, free electrons in metals rapidly absorb photon energy and become highly excited. Subsequently, this energy is transferred to the lattice through electron-phonon interactions. This electron-lattice energy exchange constitutes a unique thermal relaxation mechanism that plays a critical role in determining the metal’s response to laser exposure, particularly in the picosecond regime.

The stretched pulse duration was measured to be approximately 6 ps using an autocorrelator, as shown in Fig. 5a. Following the stretching, the laser beam was split into a probe and a pump beam using a half-wave plate and a Brewster-angle polarizer. The probe beam, transmitted through the polarizer, was directed into the ASUP module for active ultrafast imaging, while the pump beam, reflected by the polarizer, was focused onto the gold film by a lens F to induce laser-material interaction. The energy distribution between the two beams was tunable via rotation of the half-wave plate. The temporal delay between the probe and pump beams was further refined using a photodetector and oscilloscope. To ensure synchronization and effective capture of the laser-induced damage dynamics, the delay was minimized to approximately 15 ps using an optical delay line. A long-pass filter (LP) was used to block the ultraviolet and visible emissions from the laser-induced breakdown. The spectral profile of the chirped pulse, recorded by a commercial spectrometer, is shown in Fig. 5b and reveals a full width at half-maximum of 25 nm. To reconstruct spectrally resolved ultrafast images, we selected six representative wavelengths within the laser bandwidth, specifically at 1020 nm, 1025 nm, 1030 nm, 1035 nm, 1040 nm, and 1045 nm. These six spectral slices, indicated by the color bars in Fig. 5b, were used to generate six temporally distinct frames of the laser-matter interaction. With six frames mapped over a 6 ps window via a linear spectral-temporal encoding, the system achieves an effective temporal resolution of 1.2 ps per frame, yielding an ultrafast imaging frame rate of 0.83 Tfps (further discussion on the frame interval, exposure time, and cross-simulation imaging is provided in Supplementary Information Note 12). This capability allows single-shot visualization of ultrafast events on the picosecond timescale.

**Fig. 4:**
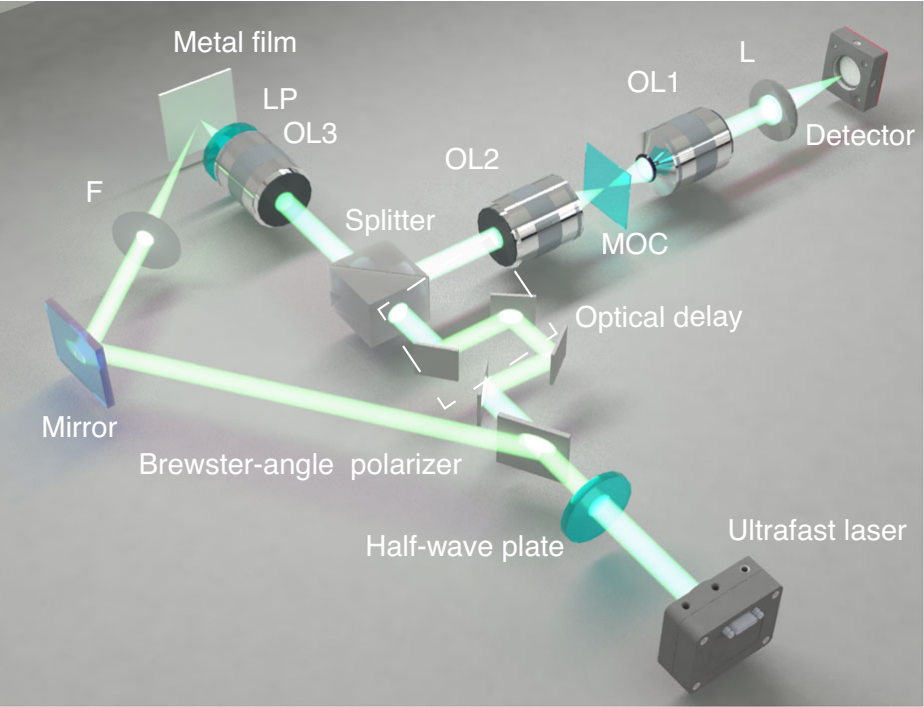
Schematic of the ASUP experimental setup. The optical delay is controlled by a translation stage to achieve different optical path lengths, thereby adjusting the time interval between the pump and probe beams. A half-wave plate combined with a Brewster-angle polarizer forms an optical attenuation assembly. The ultrafast laser is femtosecond mode-locked laser source. All other optical components are identical to those described in Fig. 3

To experimentally capture these ultrafast dynamics and validate our ultrafast photography capacity, we developed the ASUP system, as illustrated in Fig. 4. In contrast to the configuration shown in Fig. 3a, a femtosecond mode-locked laser was used as the light source. The pulse was temporally stretched using a chirped fiber Bragg grating, resulting in a chirped pulse where each wavelength corresponds to a specific time delay. This enables temporal encoding of the laser spectrum.

**Fig. 5:**
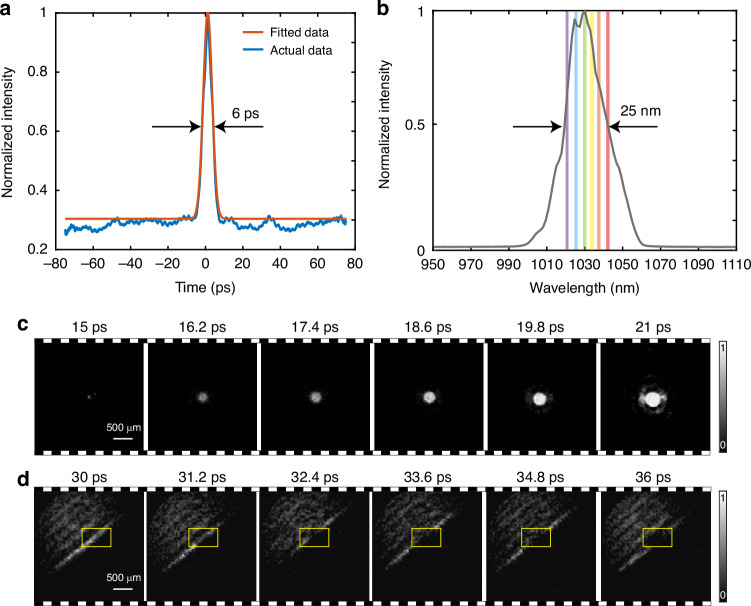
Ultrafast imaging with ASUP. **a** Temporal profile of the chirped probe pulse after pulse stretching. The blue solid line represents the measured data, while the red solid line denotes the fitted curve. **b** Spectral bandwidth of the probe light, where the six colored curves correspond to the reconstructed spectra at six selected central wavelengths. **c** Time-resolved ultrafast imaging of laser-induced dynamics on a gold (Au) film surface under femtosecond laser irradiation. **d** Evolution of the plume wavefront

Figure 5c presents a sequence of six reconstructed frames capturing the ultrafast laser-induced damage dynamics under a single-pulse fluence of 0.2 J/cm^2^. The observed evolution is in reasonable agreement with the results predicted by the classic two-temperature model (TTM), as detailed in Supplementary Information Note 6. In addition, we also recorded the dynamic process of plasma generation induced by laser irradiation. Figure 5d shows the evolution of the plume wavefront generated from the Ag film upon laser excitation, with the temporal changes highlighted in the yellow inset (detailed in Supplementary Information Note 14).

## Discussion

The results presented here establish ASUP as a compact, high-performance imaging paradigm capable of resolving transient events at an imaging speed of 0.83 Tfps with six frames in a single exposure. Unlike conventional ultrafast imaging systems that rely on bulky dispersive optics, streak cameras, or repeated pump-probe measurements, ASUP achieves pixel-level angular spectrum encoding within a nanostructured multilayer photonic chip and reconstructs ultrafast dynamics through a neural decoder enhanced with Transformer modules. This combination enables picosecond-scale temporal resolution while preserving both spectral fidelity (4 nm spectral resolution) and spatial resolution (100 μm). The convergence of RL-guided chip design with advanced computational decoding represents an alternative approach to ultrafast optical diagnostics.

### Benchmarking against existing single-shot ultrafast photography

As summarized in Supplementary Table S3, existing single-shot ultrafast photography platforms span active multiplexing schemes (e.g., STAMP, FINCOPA, H-CAP, and FISI) and passive streak camera-based or compressed sensing approaches (e.g., CUP, T-CUP, and HCUP), each achieving impressive performance by prioritizing specific metrics such as frame rate or frame number. These capabilities, however, are typically accompanied by increased optical complexity, bulky instrumentation, or specialized detectors, reflecting inherent architectural trade-offs. In contrast, ASUP adopts a chip-level design strategy that integrates temporal encoding and angular dispersion into a single multilayer dielectric element. The demonstrated 0.83 Tfps imaging with six frames thus represents a practical operating point rather than a fundamental limit. By emphasizing architectural simplicity, compactness, and scalability over metric maximization, ASUP complements existing ultrafast imaging systems and points toward more deployable and integrable implementations of single-shot ultrafast photography.

### Mechanistic implications and technical considerations

The demonstrated performance of ASUP can be attributed to a hybrid physical-computational framework, in which deterministic optical encoding is combined with data-driven reconstruction. Specifically, the MOC provides a physically grounded angular-spectrum encoding governed by its angle- and wavelength-dependent transfer function. This encoding process is deterministic and calibratable, enabling reproducible mapping between wavelength and angular spectrum at the measurement stage. The inverse design of the multilayer structure using a DQN-based reinforcement-learning strategy serves as an efficient optimization tool for navigating the large, nonconvex parameter space of thin-film stacks.

The neural network employed for decoding does not constitute an interpretable measurement model, but rather functions as a numerical inversion tool that maps the encoded BFP images to spectral intensities. The adopted residual convolutional architecture with Transformer blocks follows established design principles in image and signal reconstruction, where convolutional layers capture local features and attention mechanisms enhance global consistency. In the present work, these components are integrated and tailored to the specific angular-spectrum encoding imposed by the photonic chip, facilitating stable spectral reconstruction under the conditions investigated.

From a system-level perspective, the physical interpretability of ASUP resides primarily in the optical encoding stage, while the neural network provides a practical and flexible means of decoding the resulting high-dimensional measurements. Regarding the generalizability of the decoding network, it should be noted that the neural network is trained to invert the specific angular-spectrum encoding imposed by a given multilayer photonic chip. If a newly fabricated chip exhibits variations in its transfer function due to fabrication tolerances or design changes, the resulting BFP encoding patterns will accordingly change. In such cases, retraining or fine-tuning of the decoding network is required, as the network does not have prior knowledge of the new chip’s encoding without calibrated BFP data. This retraining reflects a numerical recalibration to the deterministic optical encoding, rather than a limitation of the ASUP framework, and the same network architecture and training pipeline can be directly reused once the new chip is characterized.

Future improvements will focus on extending the temporal frame depth by employing broader-bandwidth chirped probe pulses and further optimizing the angular encoding characteristics of the photonic chip, providing a feasible route toward increasing the frame number beyond six.

### Limitations and outlook

The present implementation of ASUP demonstrates six-frame single-shot imaging within a 6 ps temporal window, corresponding to an effective frame rate of 0.83 Tfps. This frame depth is currently limited by the available probe bandwidth and the spectral slice width required to preserve decoding fidelity. Importantly, this constraint is not intrinsic to the angular-spectrum encoding mechanism. As analyzed in the Supplementary Information Note 7, extending the probe bandwidth directly scales the accessible temporal window and frame number under the same linear time-wavelength mapping, provided that the photonic chip and decoder are adapted accordingly.

Experimentally, the demonstrations in this work focus on laser-metal interactions, which provide well-defined ultrafast signals and serve as a validation of temporal resolution and single-shot capability. While this scope does not exhaust all possible transient phenomena, additional results presented in the Supplementary Information Note 16 indicate that ASUP can also capture ultrafast reflectivity dynamics in semiconductors and wavelength-resolved biological images under suitable conditions.

The decoding process relies on a neural network trained for the specific angular-spectral transfer function of the fabricated photonic chip. Consequently, substantial fabrication-induced variations or intentional redesigns necessitate recalibration and, in some cases, retraining. Such retraining reflects the deterministic but device-specific nature of the physical encoding rather than a fundamental limitation of the approach. Moderate thickness variations primarily induce predictable spectral shifts and can be compensated through calibration.

The spatial resolution of ASUP (100 µm) is jointly constrained by the numerical aperture of the imaging optics and the finite sampling of the BFP image. Increasing NA and detector sampling can enhance both spatial and temporal fidelity, at the cost of reduced working distance or increased data throughput.

ASUP is inherently an active ultrafast imaging technique that relies on deterministic time-wavelength mapping of a chirped probe. As a result, the system is inherently suited for probe modulation processes, in which the probe wavelength is preserved while its amplitude or phase is modified by the sample. In contrast, inelastic emissions such as fluorescence or Raman scattering, which lack a fixed temporal correspondence with the probe wavelength, cannot be straightforwardly encoded within the current framework.

Overall, these limitations primarily reflect practical design choices and experimental trade-offs. The underlying chip-scale angular-spectrum encoding paradigm remains scalable and extensible, offering clear pathways toward increased frame depth, broader applicability, and improved resolution in future implementations.

## Materials and methods

### Fabrication

The multilayer photonic chip (MOC) was fabricated using ion beam deposition, employing alternating layers of tantalum pentoxide (Ta_2_O_5_) and silicon dioxide (SiO_2_). The high- and low-refractive-index materials were deposited onto a fused silica substrate (thickness of 0.1 mm) under controlled conditions to realize the desired optical interference structure. For the laser-induced damage experiments, a 100-nm-thick Au film was fabricated as the target material. The Au layer was also deposited onto a fused silica substrate via ion beam deposition. All depositions were performed in a high-vacuum environment with substrate rotation to guarantee uniformity across the surface. The high optical quality and surface smoothness of the gold film ensured its suitability for investigating ultrafast laser-matter interaction dynamics.

### Experimental setup

For the encoded angular spectrum and reconstructed ultrafast imaging experiment (Figs. 3 and 4), the laser sources include the supercontinuum laser source(YSL SC-PRO-7) with the acousto-optical tunable filter(YSL AOTF0019), and the femtosecond mode-locked laser (Yacto Technology YactoMods-FL-Ultra). Other setups include OL1 and OL2 (Nikon NPLNFL40X-0.75NA), OL3(Nikon NPLNFL20X-0.5NA), achromatic cemented lens L(LBTEK AD405-B), detector(Changchun New Industries, CN0310VIS-B), commercial spectrometer(AVANTES ULS4096CL-EVO), autocorrelator(APE pulseCheck SM Type 2), half-wave plate (Thorlabs WPH10M-1030), Brewster-angle polarizer(JCOPTIX OSPB25R-1030), high-power laser lens F with a focal length of 150 mm (Edmund Optics), and resolution target(LBTEK USAF1951 RB-N), long-pass filter LP(LBTEK MEFH10-950LP).

### ERCNN model details

An enhanced residual CNN with Transformer blocks (ERCNN) was used to decode the MOC-encoded BFP images. Each input (16 × 16 patch) was first processed by a 3 × 3 convolution followed by three residual blocks and a 2 downsampling layer to extract local features. The resulting 8 × 8 feature map (64 channels) was flattened into a 64-token sequence (token dimension = 64) and fed to a two-layer Transformer encoder with eight attention heads, hidden dimension = 64, feed forward dimension = 2048, and dropout = 0.1. A global average pooling and a fully connected layer then produced 101 spectral channels (1000–1100 nm, 1 nm sampling). Training used z-score-standardized inputs, mean-square-error loss, Adam optimizer (learning rate = 1 × 10⁻³), batch size = 50, and 1000 epochs with an 80/20 train-validation split.

## Supplementary information

Supplementary Information for Angular Spectrum-encoded Single-shot Ultrafast Photography

Dynamic process of picosecond laser-induced damage in gold films

## Data Availability

The data that support the plots in this paper are available from the corresponding authors. Source data are provided with this paper.

## Code availability

The generic code to generate the original data for all relevant tasks of this study is freely available at https://github.com/ChenHuang-Ciomp/PAPER_ULTRAFAST-PHOTOGRAPHY.
