# Supplementary material for: Angular spectrum-encoded single-shot ultrafast photography
Source: Light Sci Appl. 2026 Jun 5;15:267. doi: 10.1038/s41377-026-02289-3 (PMC13241495; doi:10.1038/s41377-026-02289-3)
Supplement: Supplementary file 1 — Supplementary Information for Angular Spectrum-encoded Single-shot Ultrafast Photography [file 41377_2026_2289_MOESM1_ESM.docx]

**Supplementary Information for Angular Spectrum-encoded Single-shot Ultrafast Photography**

Chen Huang^1,2^ ,Chunqi Jin^3✉^ ,Yi Chen^1^, Xin Zhang^1^ ,Chunrui Wang^1^ ,Hanyu Zheng^1^ , Xueqing Liu^3^ ,Qidai Chen^3^ , Junjie Sun^1✉^ , Fei Chen^1✉^

^1^ Jilin Provincial Key Laboratory of High Power Laser Technology and Application, Changchun Institute of Optics, Fine Mechanics and Physics, Chinese Academy of Sciences, 3888 Dongnanhu Road, Changchun, Jilin 130033, China

^2^ University of Chinese Academy of Sciences, Beijing 100049, China

^3^ State Key Laboratory of Integrated Optoelectronics, College of Electronic Science and Engineering, Jilin University, 2699 Qianjin Street, Changchun 130012, China

Email addresses: [huangchen22@mails.ucas.ac.cn](mailto:huangchen22@mails.ucas.ac.cn)(C. H.); chunqijin@jlu.edu.cn(C. J.); chenyi@ciomp.ac.cn(Y. C.);zhang315xin@ciomp.ac.cn(X. Z.); crwang@ciomp.ac.cn(C. W.); zhenghanyu@ciomp.ac.cn(H. Z.); [liuxueqing@jlu.edu.cn](mailto:liuxueqing@jlu.edu.cn)(X. L.); [chenqd@jlu.edu.cn](mailto:chenqd@jlu.edu.cn)(Q. C.); sunjunjie@ciomp.ac.cn(J. S.); chenfei@ciomp.ac.cn(F. C.)

Correspondence: Fei Chen (chenfei@ciomp.ac.cn); Junjie Sun (sunjunjie@ciomp.ac.cn); Chunqi Jin ([chunqijin@jlu.edu.cn](mailto:chunqijin@jlu.edu.cn))

**1. Theoretical and precise analysis of the optical transfer function of the multilayer optical chip (MOC)**

In the main text, we defined the real-space angle $\alpha$ and Fourier space angle $\psi$, and introduced the optical transfer function under p polarization. Here, we provide the detailed theoretical derivation based on temporal coupled-mode theory (TCMT). In this framework, the multilayer structure is treated as a resonator coupled to input and output ports, with external coupling rates $\gamma_{e1}(\theta)$, $\gamma_{e2}(\theta)$(external coupling rates to ports 1 and 2), and an intrinsic loss rate $\gamma_{i}$. The angle of incidence can be written as $\theta=|arcsin(\sqrt{k_{x}^{2}+k_{y}^{2}}/k_{0})|$. The transmission amplitude is expressed as^1,2^

$t^{'}\left( \omega, \theta\right)=\frac{\sqrt{\gamma_{e1}(\theta)\gamma_{e2}(\theta)}}{-i\left[ w-w_{0}\left( \theta\right) \right]+\frac{\gamma_{e1}\left( \theta\right){+\gamma}_{e2}\left( \theta\right)+\gamma_{i}}{2}}$ (S1.1)

where $w_{0}\left( \theta\right)$ is the resonance frequency that shifts with incidence angle due to changes in the in-plane wavevector. The corresponding power transmission exhibits a Lorentzian profile centered at $w_{0}\left( \theta\right)$, with a peak and bandwidth governed by the interplay between radiative coupling and intrinsic loss. As the incident angle increases, the resonance frequency typically blue-shifts and the coupling rates may vary, leading to angle-dependent spectral tuning and bandwidth control. This model provides a clear, physically intuitive explanation of how multilayer filters achieve angular selectivity through resonance behavior. When the above transmission amplitude can be achieved, we get the matrix of transmission coefficients $T\left( k_{x},k_{y} \right)=\left[ \begin{matrix} t_{s}(k_{x},k_{y}) & 0 \\ 0 & t_{p}(k_{x},k_{y}) \end{matrix} \right]$ in the basis of s and p polarization.

The optical transfer function can be expressed as when a linearly polarized light $e_{in}$ enters the MOC and exits with polarization $e_{out}$^3^

$t\left( k_{x},k_{y} \right)=e_{out}^{\mathfrak{f}}M^{-1}T(k_{x},k_{y})Me_{in}$ (S1.2)

where M is the transfer matrix from the x, y basis to the s, p basis

$M=\left[ \begin{matrix} -cos\varphi& sin\varphi\\ sin\varphi(1+\frac{\theta^{2}}{2}) & cos\varphi(1+\frac{\theta^{2}}{2}) \end{matrix} \right]$ (S1.3)

Under realistic experimental conditions, with p-polarized incident light $e_{in}=\left[ \begin{aligned} 0 \\ 1 \end{aligned} \right]$, and no restriction on output polarization, the optical transfer function is reformulated as

$$t\left( k_{x},k_{y} \right)=\left[ \begin{matrix} 1 & 1 \end{matrix} \right]\left[ \begin{matrix} -cos\varphi& \frac{sin\varphi}{1+\frac{\theta^{2}}{2}} \\ sin\varphi& \frac{cos\varphi}{1+\frac{\theta^{2}}{2}} \end{matrix} \right]\left[ \begin{matrix} t_{s}\left( k_{x},k_{y} \right) & 0 \\ 0 & t_{p}\left( k_{x},k_{y} \right) \end{matrix} \right]\left[ \begin{matrix} -cos\varphi& sin\varphi\\ sin\varphi(1+\frac{\theta^{2}}{2}) & cos\varphi(1+\frac{\theta^{2}}{2}) \end{matrix} \right]\left[ \begin{aligned} 0 \\ 1 \end{aligned} \right]$$

$=sin\varphi cos\varphi\left( tp(k_{x},k_{y})-ts(k_{x},k_{y}) \right)+{sin}^{2}\varphi t_{s}(k_{x},k_{y})+{cos}^{2}\varphi t_{p}(k_{x},k_{y})$ (S1.4)

**2. DQN inverse design of MOC**

In the main text, we formulated multilayer film design as a Markov decision process (MDP) and described the use of the deep Q network (DQN) framework for optimization. Here, we provide the detailed network design, hyperparameters (Table S1), and implementation strategies. Because the MOC inverse design is formulated as a sequential decision process, each state explicitly encodes the partial layer stack constructed up to the current step, including the material type and thickness of each deposited layer. As a result, the state space grows combinatorially with the number of layers and the discretized thickness range, rapidly reaching a scale that is infeasible to exhaustively sample or store. Moreover, consecutive design steps correspond to adjacent layers in the multilayer stack, whose optical responses are strongly correlated due to interference effects and the cumulative phase condition. Consequently, transitions collected in neighboring episodes exhibit high temporal correlation, which can bias gradient updates and degrade the stability of DQN training if all transitions are stored indiscriminately. To address these issues while maintaining sufficient exploration diversity, we adopt a constrained experience replay strategy. Specifically, the replay buffer size is limited to 200 state-action-reward tuples, and 100 transitions are randomly sampled at each training iteration (Figure S1(a)). This strategy effectively balances memory efficiency, decorrelation of training samples, and convergence stability, while preserving the ability of the DQN to identify good performance multilayer geometries.

**Table S1. Hyperparameters used for DQN-based multilayer inverse design**

| **Category** | **Hyperparameter** | **Value** |
| --- | --- | --- |
| Exploration strategy | Exploration policy | ε greedy |
|  | Initial random steps | 200 |
|  | ε | 0.1 (fixed) |
| Replay buffer | Replay buffer size | 200 |
|  | Batch size (sampled per update) | 100 |
| Training protocol | Total training episodes | 10000 |
|  | Discount factor γ | 0.95 |
|  | Optimizer | Adam |
|  | Learning rate | 0.001 |
| Q-network | Network type | Fully connected multilayer perceptron (MLP) |
|  | Hidden layers | 2 |
|  | Neurons per layer | 128 and 64 |
|  | Activation function | ReLU |
| Target network | Target network | Enabled |
|  | Update strategy | Hard update (50 steps) |
| Environment | Maximum number of layers | 15 |
|  | Thickness range | 100–300 nm |

The MOC consists of 15 alternating layers of Ta_2_O_5_ and SiO_2_, with Ta_2_O_5_ on the top. Each layer’s thickness is restricted to the 100–300 nm range. The ε‑greedy exploration parameter is set to ε = 0.1. The DQN is trained for 10,000 episodes. Figure S1(b) shows the evolution of the maximum reward versus training episodes, revealing convergence to a peak value of 0.8. Figure S1(c) compares the target transmittance of the MOC at various incidence wavelengths (left) at normal incident with the transmittance predicted by the trained DQN and the resulting layer sequence (middle).


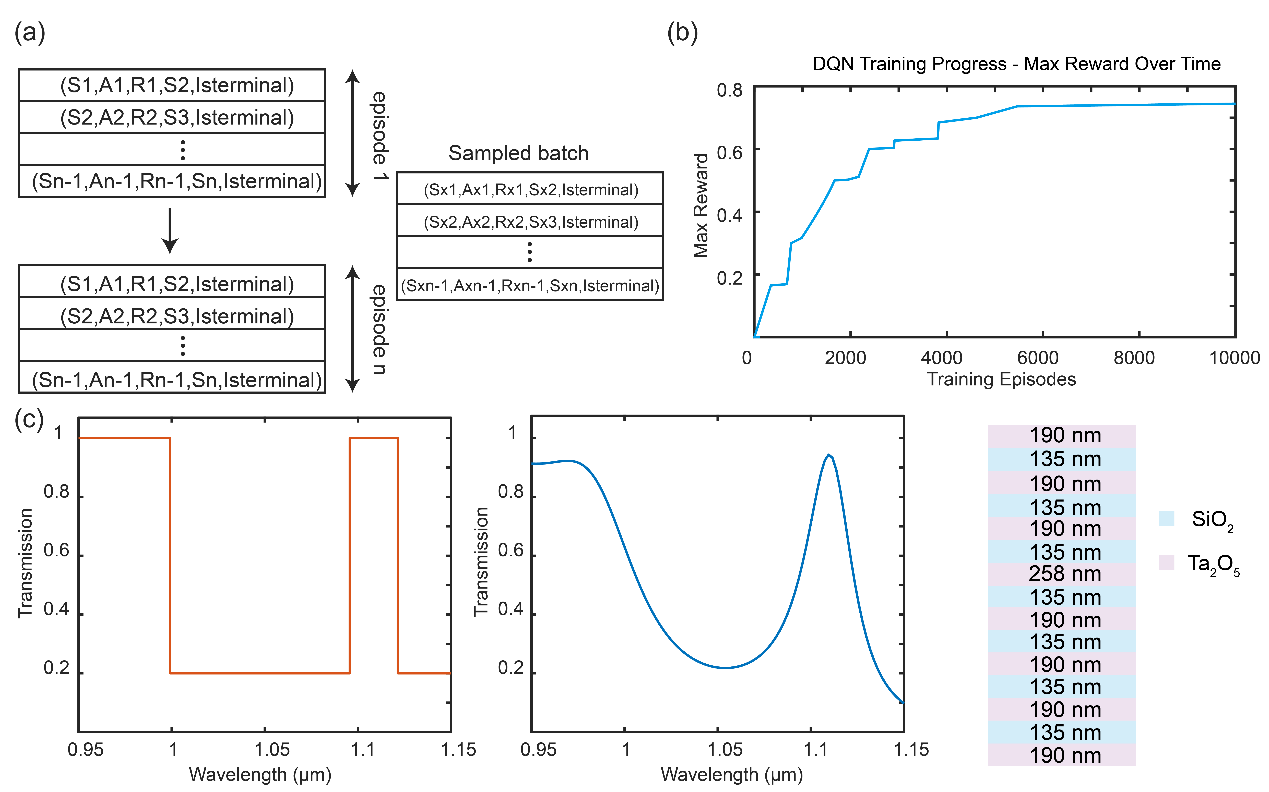


Figure S1. Workflow and Results of DQN-Based Inverse Design of MOC. (a) The process of selecting state tuples for DQN before entering neural network training. The left side shows the buffer storing episodes of the model, while the right side illustrates the random sampling of state tuples from this buffer. (b) The progression of the maximum reward achieved by DQN over 10,000 training iterations. (c) The left panel shows the target transmittance for DQN training, the middle panel presents the achieved transmittance after training, and the right panel displays the distribution of thickness and materials in the MOC.

**3. Enhanced Residual Convolutional Neural Network (ERCNN) for** **reconstructed spectrum and ultrafast image**

In the main text, we introduced the ERCNN architecture that integrates convolutional layers, residual blocks, and Transformer encoders to decode the encoded angular spectrum and reconstruct high-dimensional spectral responses. Here, we provide the detailed implementation and dataset generation process. Specifically, the ERCNN model takes the BFP images generated by the MOC under different illumination wavelengths as input, and the corresponding spectral responses as output. A dataset is generated through numerical simulations in MATLAB. Specifically, we use Gaussian-shaped spectral responses with a central wavelength $\lambda_{0}$ ranging from 1000 nm to 1100 nm in 2 nm increments, resulting in 51 samples in total. Each spectrum has a bandwidth $\sigma$ of 2 nm. The Gaussian spectral profile is defined as

$G\left( \lambda\right)=\frac{2\sqrt{ln2}}{\sigma\pi}exp(-\frac{4ln2(\lambda-\lambda_{0})}{\sigma^{2}})$ (S2.1)

Meanwhile, the simulation by equation (S2.1) also achieves 51 BFP images in total. To augment the dataset, we scaled the overall amplitude of the spectral responses, generating 30 variants per original spectrum, resulting in a total of 1530 samples. 80% of the dataset was used for training, and the remaining 20% for validation. The ERCNN model was trained for 1000 epochs, and the corresponding loss curve is shown in Figure S2. The simulation used a 160 × 160 BFP image as the original input for the pixel-level encoding and ultrafast image reconstruction section. Before training, the image was divided into patches of 16 × 16 pixels, yielding 100 sub-images in total. These sub-images were fed into the ERCNN for parallel training, resulting in 100 independently trained models, each of which outputs spectral data with n=101 channels. Finally, the outputs from all 100 models were combined to reconstruct a 10 × 10 image. Figure S3 presents the reconstructed images under four different spectral channels alongside the original spectral profile. Among them, channel 39 corresponds to the peak of the spectral curve and serves as the basis for the reconstructed image shown in the main text, Figure 2h.


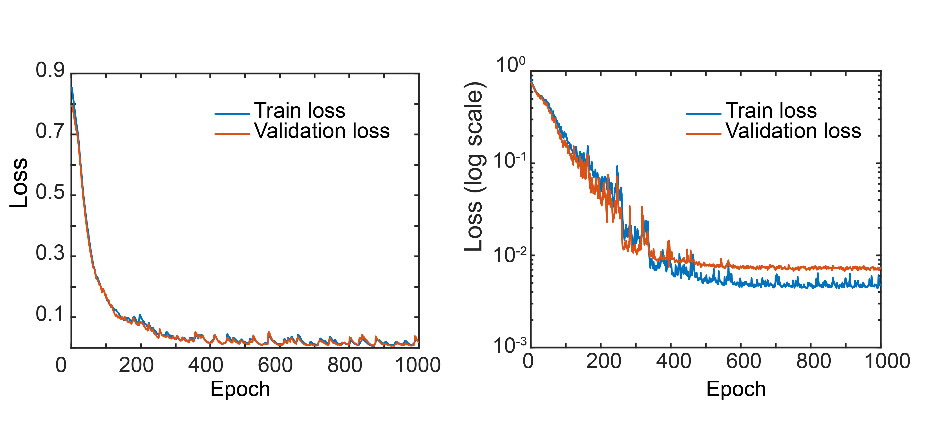


**Figure S2. The loss value of the train and validation set of simulation**. Loss evolution over 1000 epochs shown in linear scale (left) and logarithmic scale (right).


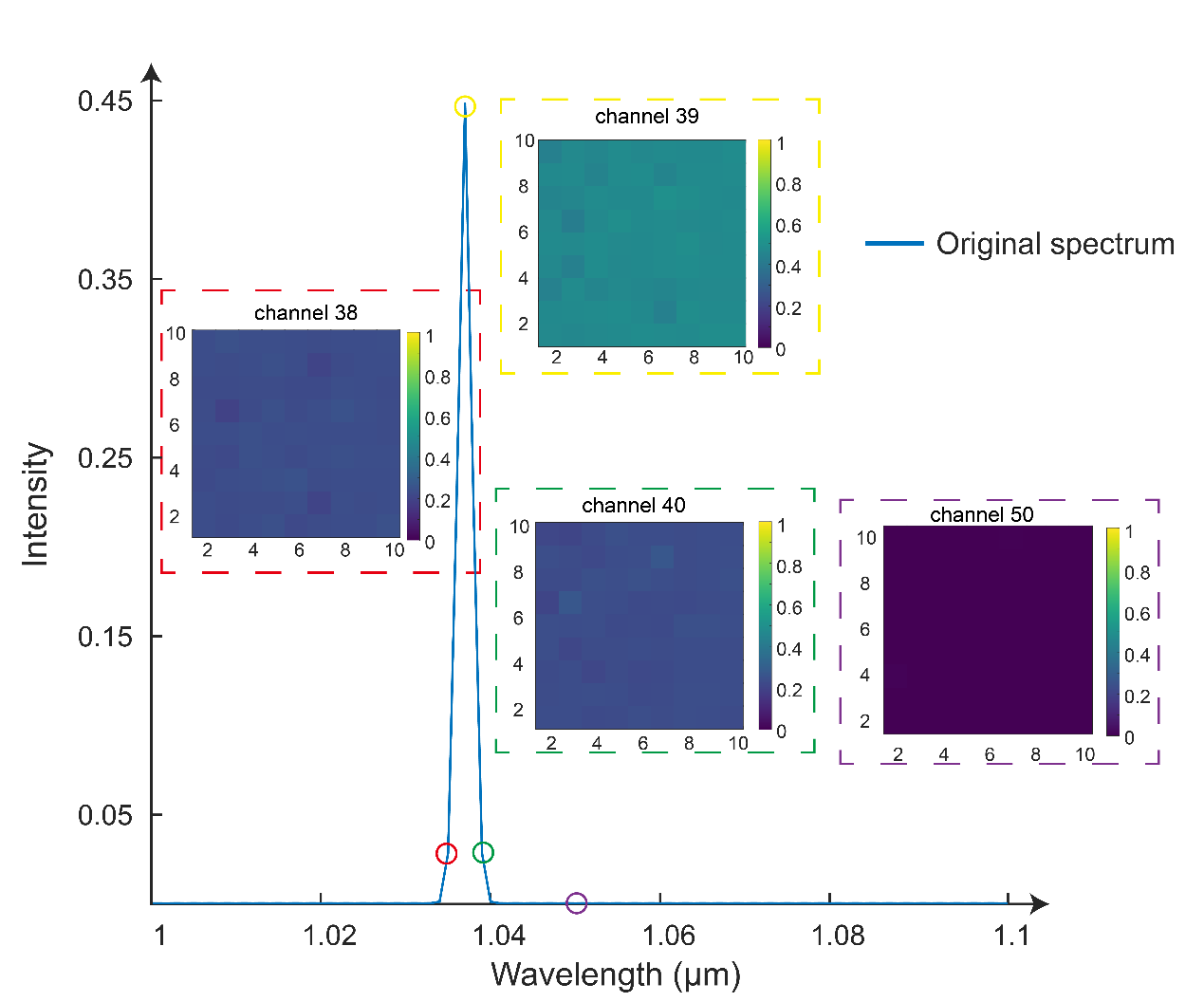


**Figure S3. Reconstructed images under four different spectral channels and original spectral curve.**

**4. Experimental encoding and decoding of angular spectrum information.**

In the main text, we demonstrated the agreement between simulated and experimentally acquired BFP images, and described the use of the ERCNN model for decoding angular spectrum information. Here, we detail the experimental workflow used for encoding and decoding. The encoding and decoding of angular spectrum information at different wavelengths are critical to the performance of our ultrafast imaging system. To enable more accurate decoding via artificial neural networks, we conducted a meticulous calibration of the encoding process. A supercontinuum laser source with an acoustic-optical tunable filter(AOTF) was used to sequentially scan monochromatic beams across a range of wavelengths. Each beam had a spectral bandwidth of 6 nm, with center wavelengths ranging from 1000 nm to 1100 nm in 1 nm increments, yielding a total of 101 distinct spectral bands. For each wavelength, we acquired the BFP image after modulation by the MOC. The spectral output remained sampled across 101 discrete channels. To further enrich the training dataset, we introduced 30 different laser power levels for each fixed wavelength, resulting in a total of 3030 sets of encoded angular spectra and corresponding spectral data. These samples were then split into a training set (80%) and a test set (20%). Each input BFP image had a spatial resolution of 2048 × 2048 pixels and was partitioned into non-overlapping 16 × 16 pixels patches, yielding 128 × 128 sub-images. These patches were used to train an ensemble of Efficient Residual Convolutional Neural Networks (ERCNNs) in parallel, producing 128 × 128 sets of independent network parameters. Each network predicted the full 101-channel spectral response. The training loss curve is shown in Figure S4.

During inference, the input BFP image is similarly divided into patches, and the corresponding trained ERCNN models are applied individually to each patch. The outputs are then aggregated to reconstruct a hyperspectral image with a spatial resolution of 128 × 128 and 101 spectral channels.


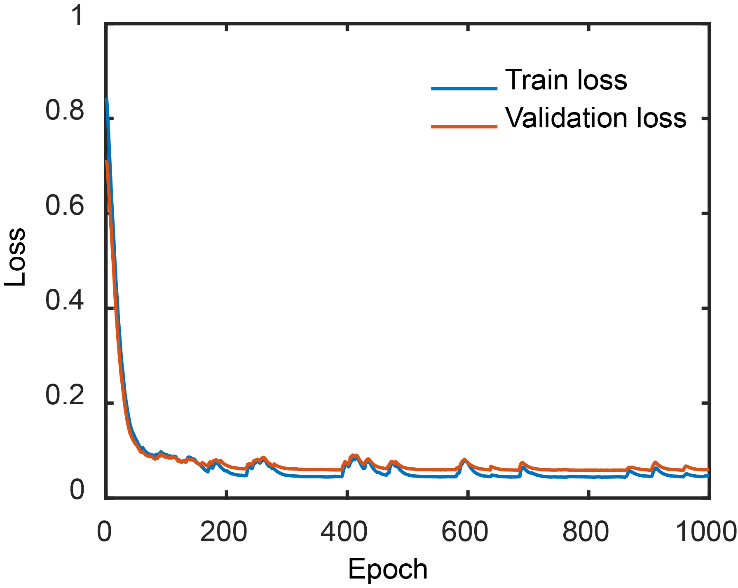


**Figure S4. The loss value of the train and validation set of experiment**.

**5. Comparisons with state-of-the-art miniaturized spectrum imaging devices and ultrafast photography**

Table S2 summarizes representative state-of-the-art miniaturized spectral imaging systems reported in recent literature, highlighting their operational wavelength ranges and spectral resolutions. For instance, a device published in Nature Photonics (2021)^6^ operates across a broad mid-infrared band (2–9 μm) with a resolution of 90 nm, suitable for molecular fingerprinting but limited in resolution. In contrast, more recent developments in Nature (2024)^7^ and Science (2022)^5^ have pushed toward higher resolution in the visible and near-infrared ranges, achieving 2.7 nm and 3 nm resolutions, respectively.

Our work focuses on the near-infrared region (1–1.1 μm), delivering a spectral resolution of 4 nm. This performance is comparable to recent achievements in Light (2024)^9^, which reported 5 nm resolution in the visible regime (0.55–0.7 μm), and surpasses earlier works such as Nat. Commun. (2022)^4^, which attained 20 nm resolution in the 1.1–1.47 μm band. Notably, our design achieves this resolution within an ultracompact footprint and is tailored for high-speed, angle-resolved spectral imaging.

**Table S2. Comparisons with state-of-the-art miniaturized spectrum imaging devices**

| **Literature** | **Operational wavelength (μm)** | **Spectral resolution (nm)** |
| --- | --- | --- |
| Nat. Commun. (2022)^4^ | 1.1-1.47 | 20 |
| Science (2022)^5^ | 0.405-0.845 | 3 |
| Nat. Photonics (2021)^6^ | 2-9 | 90 |
| Nature (2024)^7^ | 0.4-0.9 | 2.7 |
| Nat. Photonic (2023)^8^ | 0.45-0.65 | 10 |
| Light (2024)^9^ | 0.55-0.7 | 5 |
| Our work | 1-1.1 | 4 |

A quantitative comparison with representative single-shot ultrafast photography techniques is summarized in Table S3, where we report metrics that are consistently available across the literature, including imaging principle, frame rate, and spatial resolution.

From this comparison, it is evident that single-shot passive ultrafast photography methods, such as CUP-based approaches, generally achieve larger frame numbers. This advantage stems from their reliance on streak cameras and temporal shearing, which naturally enable long temporal records at the cost of bulky hardware and expensive instrumentation. In contrast, single-shot active ultrafast photography techniques—including STAMP, FINCOPA, FISI, and related methods—typically operate with a more limited number of frames, as the temporal depth is fundamentally constrained by spectral bandwidth partitioning, angular or spatial multiplexing, and optical throughput considerations. This trade-off is well recognized across the field and is consistently reflected in prior demonstrations.

Within this established context, ASUP achieves 0.83 Tfps with six frames in a single exposure, which is fully comparable to existing active single-shot ultrafast photography systems, while offering a distinctly different system architecture. Rather than employing bulk dispersive optics, echelon gratings, microlens arrays, spatial light modulators, or streak cameras, ASUP consolidates the temporal encoding and angular dispersion into a single inverse-designed multilayer dielectric photonic chip with micron-scale thickness. This monolithic component performs pixel-level angular-spectrum encoding and is inherently compatible with compact and integrated optical systems.

Although the demonstrated frame number is lower than that of some passive CUP-type approaches, ASUP distinguishes itself by achieving a substantially reduced system footprint and hardware complexit**y**, eliminating the need for streak cameras, mechanical scanning, or large-format dispersive elements. Compared with other active schemes, ASUP further reduces optical component count by replacing multiple encoding stages with a single passive photonic element.

Importantly, the presented implementation does not represent a fundamental limit of ASUP. As discussed in Supplementary Information Note 7, the frame number scales directly with the available probe bandwidth and the designed spectral slice width. With broader chirped bandwidths and optimized angular encoding orthogonality, ASUP can be extended to higher frame counts while preserving its compact, chip-based architecture.

Overall, this benchmarking clarifies that ASUP does not aim to maximize frame number and frame rate alone, but instead targets a balanced and practical operating point, achieving Tfps-class imaging with competitive temporal resolution, moderate spatial resolution, and a level of integration that is not simultaneously realized by existing single-shot ultrafast photography platforms. In this sense, ASUP introduces a complementary and conceptually new route toward deployable ultrafast imaging systems.

**Table S3. Comparisons with state-of-the-art single-shot ultrafast photography**

| **Category** | **Method** | **Imaging Frame Rate (Tfps)** | **Number of frames** | **Spatial Resolution (μm)** | **Complexity (Component count)** | **Cost**  **(Low：**  **< 10k USD**  **Medium：**  **10**–**50k USD**  **High：**  **> 50k USD)** |
| --- | --- | --- | --- | --- | --- | --- |
| Single-shot active ultrafast photography | STAMP^10^ | 4.4 | 6 | 10 | 7 | High (Driven by complex bulk-optics implementation and precision alignment requirements) |
|  | EOS^11^ | 1 | 4 | \ | 8 | Medium (Specialized EO detection modules) |
|  | PUMP^12^ | 0.08 | 16 | 35 | 6 | Medium |
|  | COFT^13^ | 256 | \ | 50 | 6 | Medium |
|  | CSMUP^14^ | 0.25 | 25 | 0.83 | 9 | High |
|  | FINCOPA^15^ | 15 | 4 | 12 | 8 | High |
|  | FDT^16^ | 0.42 | 5 | 20 | 7 | High (Driven by complex multi-angle optical geometry) |
|  | FTOP^17^ | 0.25 | 4 | \ | 5 | Medium |
|  | LIF^18^ | 10 | 7 | 79 | 6 | Medium |
|  | FRAME^19^ | 5 | 4 | 66 | 5 | Medium |
|  | TSFM^20^ | 4.8 | 14 | 8 | 7 | High (SLM, custom echelon and interferometric stability requirements) |
|  | FISI^21^ | 12.6 | 6 | 9.2 | 6 | Medium |
|  | SF-STAMP^22,23^ | 7.5 | 25 | \ | 6 | Medium (Simpler than STAMP; DOE and filters dominate cost)) |
|  | OPR^24^ | 2 | 12 | 5.5 | 7 | Medium |
|  | CUST^25^ | 3.85 | 60 | \ | 7 | Medium |
|  | H-STAR^26^ | 5.52 | 120 | 4.9 | 7 | Medium |
|  | SCARF^27^ | 156.3 | 132 | \ | 7 | Medium |
|  | H-CAP^28^ | 0.74 | 120 | 55.7 | 6 | Medium |
| Singles-shot passive ultrafast photography | CUP^29^ | 0.1 | 350 | 3000 | 8 | High (Streak camera) |
|  | fsLS-CUP^30^ | 0.25 | 220 | 25 | 5 | Medium |
|  | SP-CUP^31^ | 0.25 | 300 | \ | 5 | High (Streak camera and polarization-stereo multiplexing) |
|  | UV-CUP^32^ | 0.5 | 1500 | 99 | 7 | High (streak camera and photocathode fabrication) |
|  | HCUP^33^ | 0.5 | \ | 305 | 9 | High (Streak camera and hyperspectral dispersion) |
|  | T-CUP^34^ | 10 | 350 | \ | 5 | Medium |
|  | LLE-CUP^35^ | 0.1 | 300 | \ | 6 | Medium |
|  | LIFT^36^ | 0.5 | >1000 | \ | 8 | High |
| Our work | ASUP | 0.83 | 6 | 100 | 4 | Low (no streak camera, SLM/DMD, nonlinear crystals, or multi-camera synchronization is required) |

**6. Theoretical analysis based on the two-temperature model (TTM) for ultrafast laser irradiation metal film**

In the main text, we compared the experimentally observed ultrafast damage dynamics with predictions of the TTM. Here, we present the theoretical framework and detailed simulation procedure. The interaction between ultrafast laser pulses and metals can be quantitatively described by solving the TTM equations (S6.1 and S6.2), which capture the temporal evolution of electron and lattice temperatures on the picosecond timescale.

$C_{e}\left( T_{e} \right)\frac{\partial T_{e}}{\partial t}=\nabla\cdot\left( K_{e}\left( T_{e},T_{l} \right)\nabla T_{e} \right)-G(T_{e})\left( T_{e}-T_{l} \right)+S$ (S6.1)

$C_{l}\left( T_{l} \right)\frac{\partial T_{l}}{\partial t}=\nabla\cdot\left( K_{l}\left( T_{e},T_{l} \right)\nabla T_{l} \right)+G(T_{e})(T_{e}-T_{l})$ (S6.2)

In this model, $T_{e}$ and $T_{l}$ represent the electron temperature and the lattice temperature, respectively. $C_{e}$ and $C_{l}$ represent the specific heat capacities of the electrons and the lattice, respectively, while $K_{e}$ and $K_{l}$ are the thermal conductivity of the electrons and the lattice. $G$ denotes the electron-photon coupling coefficient, and $S$ is the source term. Lattice thermal melting is considered through the inclusion of latent heat, with the lattice thermal conductivity set to one percent of the electronic thermal conductivity. The electronic thermal conductivity varies with the electron temperature and is expressed as $K_{e}$=$K_{e0}(\frac{B_{e}T_{e}}{A_{e}\left( T_{e} \right)^{2}+B_{e}T_{l}})$. Both the electron-phonon coupling coefficient and electronic thermal melting parameters are determined based on first-principles calculations. The simulation parameters are summarized in Table S4.

**Table S4. Parameters of Au in TTM**

| $C_{l}$ [Jm^-3^K^-1^] | $C_{l}\left( T_{l} \right)=C_{l0}+\frac{L_{f}}{10\sqrt{\pi}}exp[-\left( \frac{T_{l}-T_{m}}{10} \right)^{2}]$ |
| --- | --- |
| $C_{e}$ [Jm^-3^K^-1^] | First-principles ^37^ |
| $K_{e}$ [Wm^-1^K^-1^] | $318\left( \frac{B_{e}T_{e}}{A_{e}\left( T_{e} \right)^{2}+B_{e}T_{l}} \right) ADDIN ZOTERO\_ITEM CSL\_CITATION \{"citationID":"A5RZf9N7","properties":\{"formattedCitation":"\backslash\backslash super 38\backslash\backslash nosupersub\{\}","plainCitation":"38","noteIndex":0\},"citationItems":[\{"id":9606,"uris":["http://zotero.org/users/9390588/items/SJUVWEPH"],"itemData":\{"id":9606,"type":"article-journal","abstract":"The employment of femtosecond pulsed lasers has received significant attention due to its capability to facilitate fabrication of precise patterns at the micro- and nano- lengths scales. A key issue for efficient material processing is the accurate determination of the damage threshold that is associated with the laser peak fluence at which minimal damage occurs on the surface of the irradiated solid. Despite a wealth of previous reports that focused on the evaluation of the laser conditions that lead to the onset of damage, the investigation of both the optical and thermal response of thin films of sizes comparable to the optical penetration depth is still an unexplored area. In this report, a detailed theoretical analysis of the impact of various parameters such as the photon energies and material thickness on the damage threshold for various metals (Au, Ag, Cu, Al, Ni, Ti, Cr, Stainless Steel) is investigated. A multiscale physical model is used that correlates the energy absorption, electron excitation, relaxation processes and minimal surface modification. The satisfactory agreement of the theoretical model with some experimental results indicates that the damage threshold evaluation method could represent a systematic approach towards designing efficient laser-based fabrication systems and optimizing the processing outcome for various applications.","container-title":"Optics \& Laser Technology","DOI":"10.1016/j.optlastec.2022.108484","ISSN":"00303992","journalAbbreviation":"Optics \& Laser Technology","language":"en","page":"108484","source":"DOI.org (Crossref)","title":"Damage threshold evaluation of thin metallic films exposed to femtosecond laser pulses: The role of material thickness","title-short":"Damage threshold evaluation of thin metallic films exposed to femtosecond laser pulses","volume":"156","author":[\{"family":"Tsibidis","given":"G.D."\},\{"family":"Mansour","given":"D."\},\{"family":"Stratakis","given":"E."\}],"issued":\{"date-parts":[["2022",12]]\},"citation-key":"tsibidisDamageThresholdEvaluation2022"\}\}],"schema":"https://github.com/citation-style-language/schema/raw/master/csl-citation.json"\}$^38^ |
| $K_{l}$ [Wm^-1^K^-1^] | $0.01K_{e} ADDIN ZOTERO\_ITEM CSL\_CITATION \{"citationID":"vm1DHMwD","properties":\{"formattedCitation":"\backslash\backslash super 39,40\backslash\backslash nosupersub\{\}","plainCitation":"39,40","noteIndex":0\},"citationItems":[\{"id":10151,"uris":["http://zotero.org/users/9390588/items/KT98MMJ6"],"itemData":\{"id":10151,"type":"article-journal","abstract":"The heating processes of the single-layer gold thin ﬁlm and the two-layer ﬁlm assembly of gold padded with other metal (silver, copper and nickel) irradiated by femtosecond laser pulse are studied by the two-temperature model. It is found that the substrate metal can change energy transport, which is corresponding to the temperature changing process, and the thermal equilibrium time. Compared with the single-layer gold ﬁlm at the same laser ﬂuence, the two-layer ﬁlm structure can change the damage threshold of the gold surface. Our results indicate that we can maximize the damage threshold of the gold ﬁlm surface by altering the thickness ratio of the gold layer and the substrate layer in the two-layer ﬁlm assembly.","container-title":"Applied Surface Science","DOI":"10.1016/j.apsusc.2010.08.122","ISSN":"01694332","issue":"5","journalAbbreviation":"Applied Surface Science","language":"en","page":"1678-1683","source":"DOI.org (Crossref)","title":"Modeling of femtosecond laser damage threshold on the two-layer metal films","volume":"257","author":[\{"family":"Chen","given":"A.M."\},\{"family":"Xu","given":"H.F."\},\{"family":"Jiang","given":"Y.F."\},\{"family":"Sui","given":"L.Z."\},\{"family":"Ding","given":"D.J."\},\{"family":"Liu","given":"H."\},\{"family":"Jin","given":"M.X."\}],"issued":\{"date-parts":[["2010",12]]\},"citation-key":"chenModelingFemtosecondLaser2010"\}\},\{"id":10150,"uris":["http://zotero.org/users/9390588/items/FPULEM65"],"itemData":\{"id":10150,"type":"article-journal","container-title":"Thin Solid Films","DOI":"10.1016/j.tsf.2012.06.027","ISSN":"00406090","journalAbbreviation":"Thin Solid Films","language":"en","license":"https://www.elsevier.com/tdm/userlicense/1.0/","page":"209-216","source":"DOI.org (Crossref)","title":"Ultrafast investigation of electron dynamics in the gold-coated two-layer metal films","volume":"529","author":[\{"family":"Chen","given":"Anmin"\},\{"family":"Sui","given":"Laizhi"\},\{"family":"Shi","given":"Ying"\},\{"family":"Jiang","given":"Yuanfei"\},\{"family":"Yang","given":"Dapeng"\},\{"family":"Liu","given":"Hang"\},\{"family":"Jin","given":"Mingxing"\},\{"family":"Ding","given":"Dajun"\}],"issued":\{"date-parts":[["2013",2]]\},"citation-key":"chenUltrafastInvestigationElectron2013"\}\}],"schema":"https://github.com/citation-style-language/schema/raw/master/csl-citation.json"\}$^39,40^ |
| $G$ [Wm^-3^K^-1^] | First-principles ^37^ |
| $C_{l0}$ [×10^6^ Jm^-3^K^-1^] | 2.48 ^38^ |
| $L_{f}$ [kJ kg^-1^] | 64 |
| $T_{m}$ [K] | 1337 |
| $B_{e}$ [s^-1^K^-1^] | 1.25 ×10^11 38^ |
| $A_{e}$ [s^-1^K^-2^] | 1.18 ×10^7 38^ |
| $S$ | $\sqrt{\frac{4ln2}{\pi}}(1-R)\frac{J}{\tau}exp(-4ln2\frac{{(t-t_{0})}^{2}}{\tau^{2}})e^{-\alpha z}$ |
| J [J cm^-2^] | 0.2 |
| $\tau$ [ps] | 6 |

Building on the foregoing modeling, we obtain the temporal evolution of the electron and lattice temperatures in a metal film under ultrafast laser excitation. Because melting is directly governed by the lattice temperature, we report only the lattice-temperature dynamics. Figure S5 juxtaposes the lattice-temperature trajectory with the time-resolved average intensity in the central region of the ultrafast images shown in Figure 5 of the main text, revealing a high degree of concordance. This agreement indicates that the ASUP ultrafast measurement system provides a reliable characterization of the transient dynamics of laser-induced damage and the electron-lattice nonequilibrium governed by the classical TTM equations.


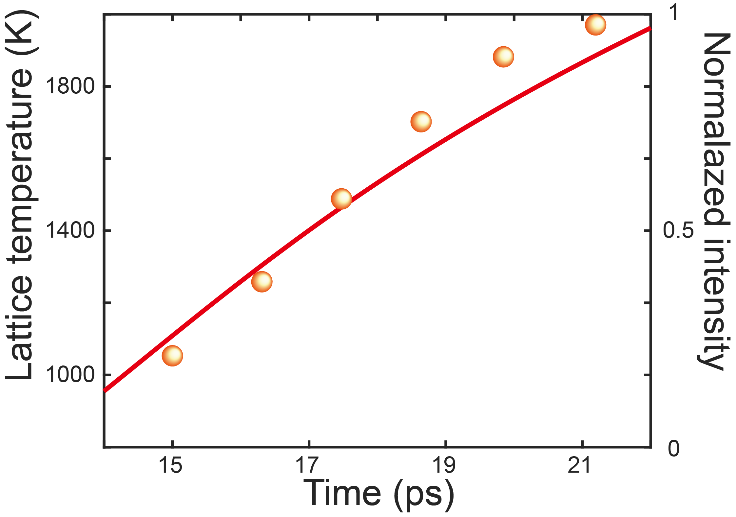


**Figure S5. Temporal evolution of the lattice temperature and the mean intensity of the image frames.** The red solid line represents the lattice-temperature dynamics, while the orange spheres denote the average intensity values of the central regions across different ultrafast images.

**7. Quantitative evaluation of bandwidth-depth scalability**

To address the trade-off between imaging depth and bandwidth, we simulated the performance of the proposed system when the spectral bandwidth is extended from the experimental 25 nm case to 50 nm and 100 nm. The experimentally demonstrated baseline uses a 25 nm probe bandwidth that is linearly stretched to 6 ps (Figure 5 of the main text), implying a time-wavelength slope α = T/Δλ = 6 ps / 25 nm = 0.24 ps/nm. The device-limited per frame spectral slice is set to Δλ_slice_ = 4 nm (Figure 3e), and the encoder/dispersion remain unchanged. Frames are formed by non‑overlapping slices centered within the working band. For wider bands, the center is shifted to 1050 nm so that the 50 nm and 100 nm cases remain inside the 1000–1100 nm design window of the MOC and the decoder. With linear mapping and non‑overlapping Δλ_slice_, the total temporal window and frame count scale as T = α Δλ, N = |Δλ / 5nm + 1|. Two temporal figures are reported: (i) center spacing Δt_center = α Δλ_slice_ = 0.96 ps; (ii) sequence spacing Δt_seq = T/(N − 1), relevant as an effective per frame step across a fixed window.

Table S5 | Predicted scalability with bandwidth.

| Δλ (nm) | Center λ (nm) | Frames N | Effective encoding window T (ps) | Δt_center (ps) | Δt_seq (ps) |
| --- | --- | --- | --- | --- | --- |
| 25 | 1030 | 6 | 6.0 | 0.96 | 1.20 |
| 50 | 1050 | 11 | 12.0 | 0.96 | 1.20 |
| 100 | 1050 | 21 | 24.0 | 0.96 | 1.20 |

We simulated the complete encoding-decoding process using the experimentally validated MOC transfer function and the same ERCNN architecture described in the main text. The spectra were uniformly sampled at 1 nm intervals across the 1000–1100 nm range, and 4 nm spectral slices were used to define individual temporal frames according to the linear dispersive mapping. Figure S6 presents the reconstructed results and quantitative errors for the three bandwidth cases (25 nm, 50 nm, and 100 nm). The results show that, with bandwidth-aware retraining, the reconstruction accuracy for the 50 nm and 100 nm cases exhibits only modest degradation compared with the 25 nm baseline, confirming the scalability of the proposed system.


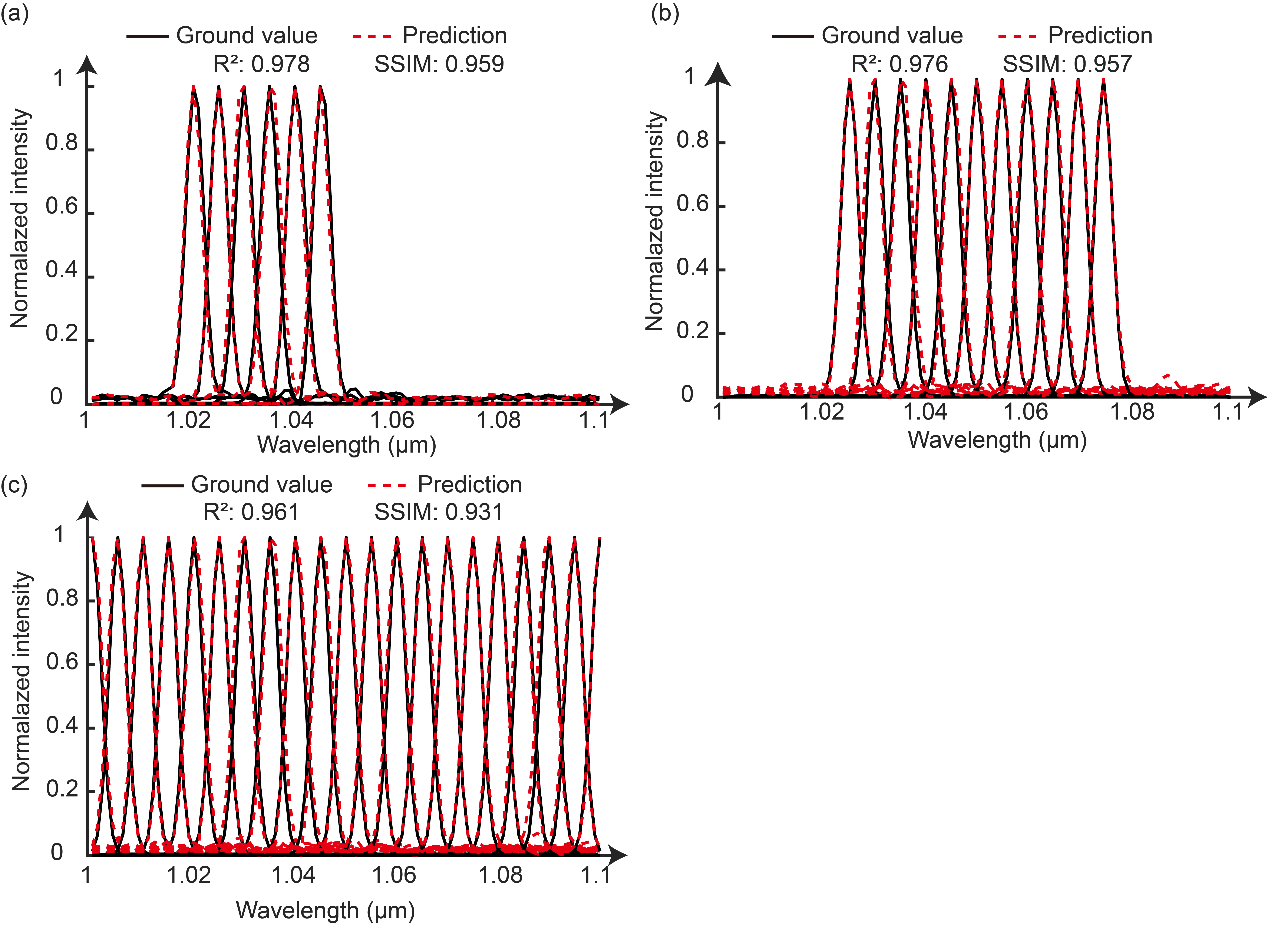


**Figure S6. Reconstructed spectra and quantitative errors for the three bandwidth conditions.** The spectral profiles predicted by the ERCNN model (red dashed lines) are compared with the ground-truth spectra (solid lines). Panels (a), (b), and (c) correspond to reconstructions with bandwidths of 25 nm, 50 nm, and 100 nm, respectively.

**8. Correlation coefficients calculation and analysis of the unique dependences**

To quantitatively evaluate the angular dispersion behavior of the MOC, we calculated the correlation coefficients between the transmission spectra obtained at different incident angles. The correlation coefficient between two angles θᵢ and θⱼ is defined as

$\rho_{ij}=\frac{\sum_{\lambda} (T(\lambda,\theta_{i})-T_{i})(T(\lambda,\theta_{j})-T_{j})}{\sqrt{\sum_{\lambda} (T(\lambda,\theta_{i})-T_{i})^{2}\sum_{\lambda} (T(\lambda,\theta_{j})-T_{j})^{2}}}$ (S 8.1)

where $T(\lambda,\theta)$denotes the transmittance at wavelength λ and incidence angle θ, and $T_{i/J}$ is the mean transmittance over the wavelength range for θᵢ_/j_. All spectra were simulated using the transfer-matrix method (TMM) across 1000 – 1100 nm with angular increments. The simulation was performed using the TMM for p-polarization with incidence angles from 0° to 48.59° (this angle corresponds to 0.75 NA) in 0.5° increments. Figure S7(a) shows the resulting correlation matrix. The map exhibits a diagonally elongated band of high correlation ($\rho$ = 1), which gradually decays as the angular difference increases. This indicates a continuous and monotonic spectral shift of the resonance peak with incident angle rather than abrupt changes. Such a structured correlation pattern reveals that neighboring angular channels share overlapping spectral features but evolve predictably, confirming the presence of a well-behaved dispersive interface. The absence of random or fragmented correlation patches demonstrates that the MOC introduces deterministic angular dispersion governed by the designed multilayer interference. This smooth, nearly linear decorrelation ensures that each incidence angle corresponds to a distinct yet spectrally connected transmittance profile, a prerequisite for stable angular-spectral encoding. The diagonal correlation pattern therefore signifies that the MOC achieves an optimal angle-dependent state—balancing sufficient spectral separation for encoding while maintaining continuous angular tunability, which facilitates robust calibration and decoding.

To further clarify the optimal angle-dependent behavior of the MOC, we analyzed the unique dependences of the angular-spectrum-domain transmission mapping. Building on the transfer function in (S1.4), we quantify how each spectral channel produces a distinct angular-spectrum pattern by using an L1 mapping-difference metric over the NA-limited region:

$D(\lambda_{i},\lambda_{j})=\sum_{k_{x},k_{y}} \mid\text{ }I\left( k_{x},k_{y},\lambda_{i} \right)-I\left( k_{x},k_{y},\lambda_{j} \right)\text{ }\mid, I(k_{x},k_{y},\lambda)=\mid t(k_{x},k_{y},\lambda)\mid^{2}$ (S 8.2)

To remove trivial throughput bias, each $I(k_{x},k_{y},\lambda)$ is normalized by its masked mean before evaluating $D$. Figure S7(b) shows the $D(\lambda_{i},\lambda_{j})$ heatmap across 1000–1100 nm. As expected, the diagonal is near zero and $D$ increases with $\mid\lambda_{i}-\lambda_{j}\mid$. Beyond this generic trend, the map exhibits banded off-diagonal ridges whose locations reflect the multilayer-interference dispersion of the MOC: wavelength pairs that straddle faster angular shifts yield larger $D$, whereas pairs within slowly varying regions produce smaller $D$. Figure S7(c) plots the nearest-neighbour distinctiveness $D_{\mathrm{NN}}(\lambda)=\min_{\lambda^{'}\neq\lambda}D(\lambda,\lambda^{'})$. We observe a monotonic rise of $D_{\mathrm{NN}}$ across most of the band with a shallow local dip around the mid-band, followed by a pronounced increase toward 1.09–1.10 µm. This indicates that adjacent spectral channels become progressively easier to discriminate toward the long-wavelength end, while the mid-band represents a gentler dispersion region. Both behaviors are consistent with the designed angle-dependent phase condition of the multilayer stack and our measured/simulated BFP trends in the main text


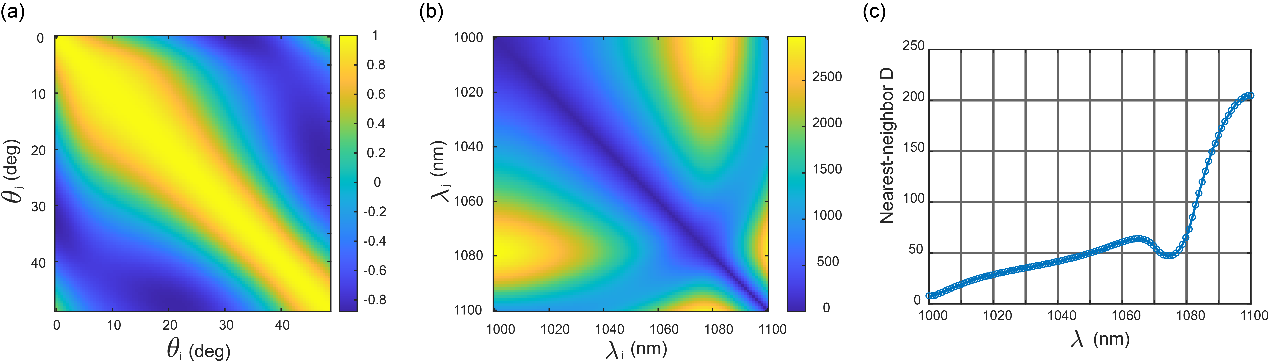


**Figure S7.** (a) Correlation-coefficient matrix of $T(\lambda,\theta)$. (b) Angular-spectrum mapping-difference heatmap $D(\lambda_{i},\lambda_{j})$computed from $I=\mid t\mid^{2}$of (S1.4) within the NA-limited region. (c) Nearest-neighbor distinctiveness $D_{\mathrm{NN}}(\lambda)$, revealing band-dependent discriminability with a strong rise near 1.09–1.10 µm.

The joint evidence from (i) structured angle-spectrum correlation and (ii) high, band-resolved mapping-difference demonstrates that our MOC operates in an optimal angle-dependent regime: spectral channels generate unique and well-separated angular-spectrum patterns while preserving smooth, calibratable evolution with angle. This regime maximizes encoding separability without sacrificing deterministic tunability—exactly the trade-off enforced by our inverse-designed multilayer under fabrication and throughput constraints. Consequently, the ERCNN can learn a stable one-to-one decoding from BFP images to spectra.

**9. Simulations of temporal distortion under different NAs**

The proposed method relies on the mapping relationship among the angular spectrum, wavelength, and time. Consequently, variations in the angular spectrum domain—represented by different numerical apertures (NAs)—can influence spectral reconstruction and introduce temporal distortions. To analyze the effect of NA on temporal accuracy, we simulated the spectral reconstruction under different NA conditions. The reconstruction settings and spatial resolution were kept identical to those described in Section 3 of the Supplementary Information. Figure S8(a–c) compares NA = 0.3, 0.5, and 0.75. Reducing NA restricts the high $k$ support and suppresses angular components that carry essential temporal information, which increases spatial-temporal cross-talk and degrades reconstruction. Quantitatively, the correlation coefficient improves from SSIM $=0.704$ and $R^{2}=0.465$ at NA = 0.3 to SSIM $=0.893$ and $R^{2}=0.916$ at NA = 0.5, and SSIM $=0.962$ and $R^{2}=0.987$ at NA = 0.75. These results indicate that higher NA mitigates temporal distortion by enlarging the usable angular-spectrum bandwidth.


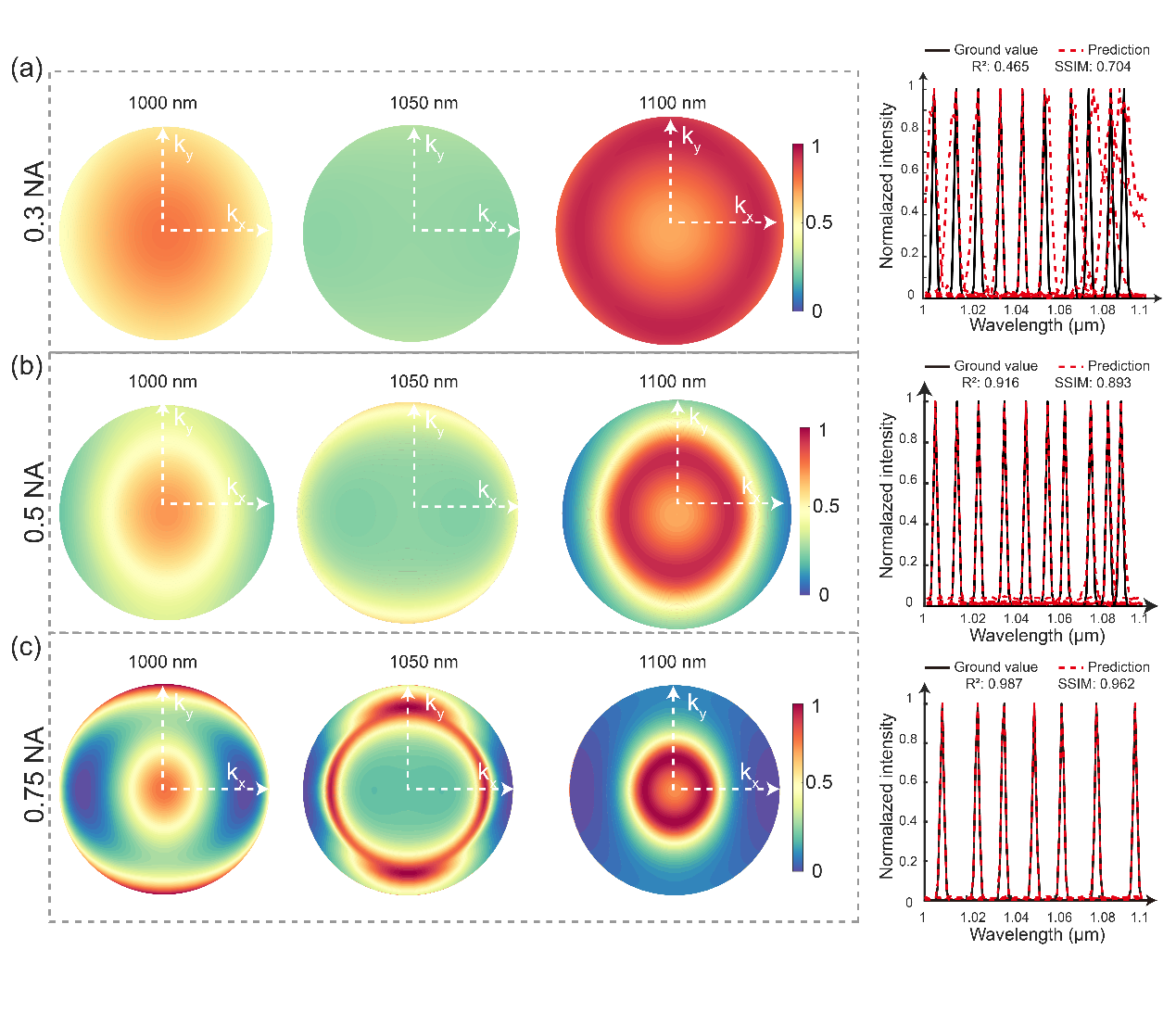


**Figure S8. Effect of numerical aperture on spatio-temporal fidelity.** For NA = 0.3 (a), 0.5 (b), and 0.75 (c), the left triplets show the normalized angular-spectrum transmission maps over $(k_{x},k_{y})$at 1000, 1050, and 1100 nm, and the right panels compare ERCNN-predicted spectra (red dashed) with ground truth (black solid).

**10. Effect of intermediate-layer thickness on the MOC and calibration under sample-induced variations**

The MOC structure used in this work (see Figure S1) can be regarded as a Fabry-Pérot-like multilayer system, where two outer DBR stacks serve as mirrors and a central Ta_2_O_5_ spacer forms the cavity. In this configuration, the resonance wavelength is mainly determined by the optical thickness of the spacer layer, while the DBR mirrors primarily define the finesse and remain relatively robust to small parameter changes. To evaluate the influence of the spacer thickness, we simulated the normal-incidence transmission spectra for different thickness values. As shown in Figure S9, increasing the spacer thickness leads to a clear red-shift of the resonance band, confirming that the cavity thickness directly controls the spectral position of the encoding region.

When the sample itself introduces refractive-index gradients or thickness variations, the optical transfer function of the system becomes spatially variant, resulting in a local shift of the resonance wavelength and, consequently, a modified wavelength-time mapping. In practice, such perturbations primarily act as wavelength offsets rather than distortions of the overall transfer shape. Therefore, the calibration strategy can be readily adapted by updating the wavelength-thickness correspondence obtained either from simulation or reference measurement. Once the local resonance shift is determined, the wavelength-time lookup table can be recalibrated by substituting $\lambda^{'}=\lambda-\Delta\lambda(x,y)$, where $\Delta\lambda(x,y)$ represents the local spectral offset arising from index or thickness variations. In experiments, this adjustment can be achieved by illuminating the device with a broadband reference and fitting the local resonance position, or by slightly shifting the encoded spectral window and illumination band to recenter the operational ridge. Overall, variations in thickness or refractive index mainly shift the resonance position without changing the intrinsic mapping behavior of the MOC. By recalibrating the wavelength-time correspondence or adjusting the working spectral range, the encoding-decoding process can maintain temporal fidelity even when the sample introduces moderate spatial inhomogeneities.


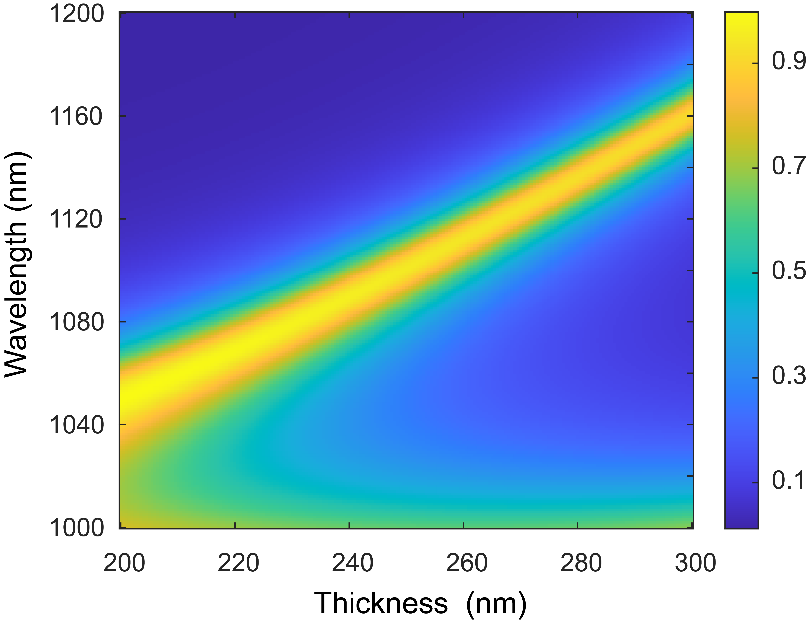


**Figure S9. Thickness-dependent resonance of the MOC at normal incidence.** Simulated transmission map as a function of spacer thickness (200–300 nm) and wavelength (1000–1200 nm).

**11. Analysis of inherent imaging capabilities**

The inherent spatial resolution of the imaging system in this study was evaluated under static object conditions. The USAF 1951 resolution target (shown in Figure S10(a), left) was used as the imaging object. The reconstructed spectral images from this system are capable of resolving objects down to 100 µm, as indicated by the resolution target in region 2-3 on the left side of Figure S10(a). The right side of Figure S10(a) shows the image before MOC reconstruction, which serves as a reference for comparison with the reconstructed and ground-truth images.

To quantify the joint impact of MOC encoding and CNN decoding on imaging performance, we present the results at different epochs of the training process in Figure S10(b). As the number of training epochs increases, the image reconstruction quality improves, with clearer and sharper features becoming apparent. This demonstrates the effectiveness of the iterative training process in enhancing imaging resolution and overall performance. Code availability is provided in the main text.


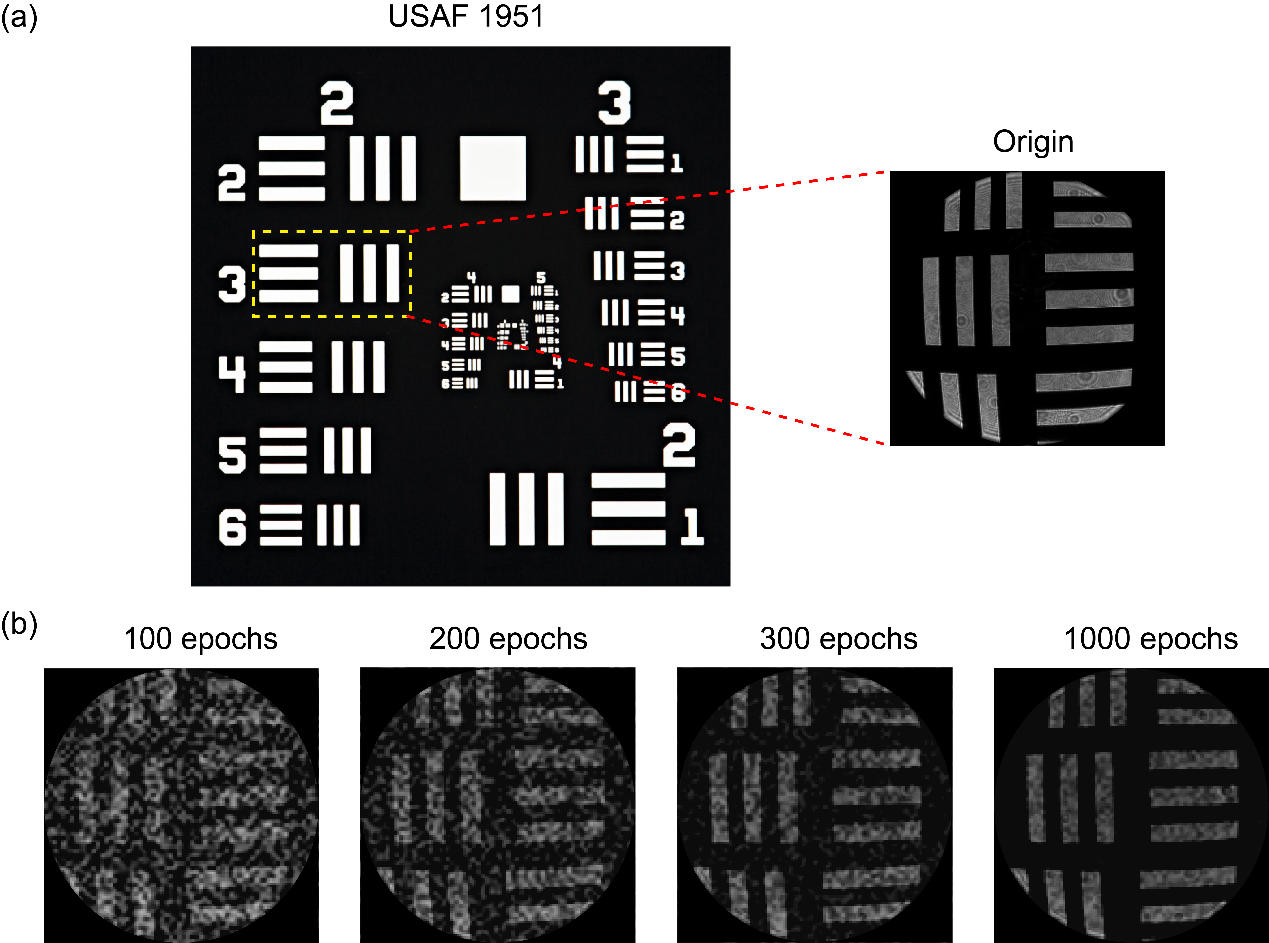


**Figure S10. Inherent imaging capabilities of the system.** (a) USAF 1951 resolution target used for evaluating imaging performance, with the left side showing the target and the right side depicting the image before MOC reconstruction. (b) Training progress with CNN decoding at different epochs (100, 200, 300, 1000 epochs).

**12. Temporal mapping characteristics:** **frame interval, exposure time, and cross-simulation imaging**

In ultrafast imaging, the interplay between frame interval and exposure time determines whether adjacent frames are temporally isolated or partially overlapped. Following conventions in high-speed photography and STAMP^10^, the exposure time is defined as the FWHM of the intensity envelope of the effective time window, whereas the frame interval is the temporal separation between the centers of consecutive spectral slices. For the experimentally demonstrated 25 nm bandwidth stretched to 6 ps. With a slice width of Δλ_slice_ = 4 nm, the exposure time is 0.96 ps. The frame interval is 1.2ps, corresponding to an effective frame rate of 0.83 Tfps. These quantities and their relationships are summarized in Figure S11(a). When the exposure time is shorter than the interval, frames record non-overlapping temporal information. As the exposure approaches or exceeds the interval, partial temporal overlap occurs, leading to correlated content between neighboring frames. A controlled degree of overlap (approximately 8% in our experiment) preserves smooth temporal continuity while avoiding excessive blurring. This balance between separation and continuity defines an optimal operating regime for high-fidelity ultrafast movie reconstruction. To visualize the effect, we performed cross-simulation imaging. Figure S11(b) plots the inter-frame mixing matrix, which is strongly diagonal with small nearest-neighbor terms reflecting the 8% overlap. Figure S11(c) shows six original microscopic frames (containing sperm cells, yeast cells, and a motile vinyl worm-like organism; yellow circles mark its positions and trajectory) and the corresponding simulation-based reconstructions. The designed overlap does not introduce noticeable artifacts, and both temporal continuity and spatial details are well preserved.


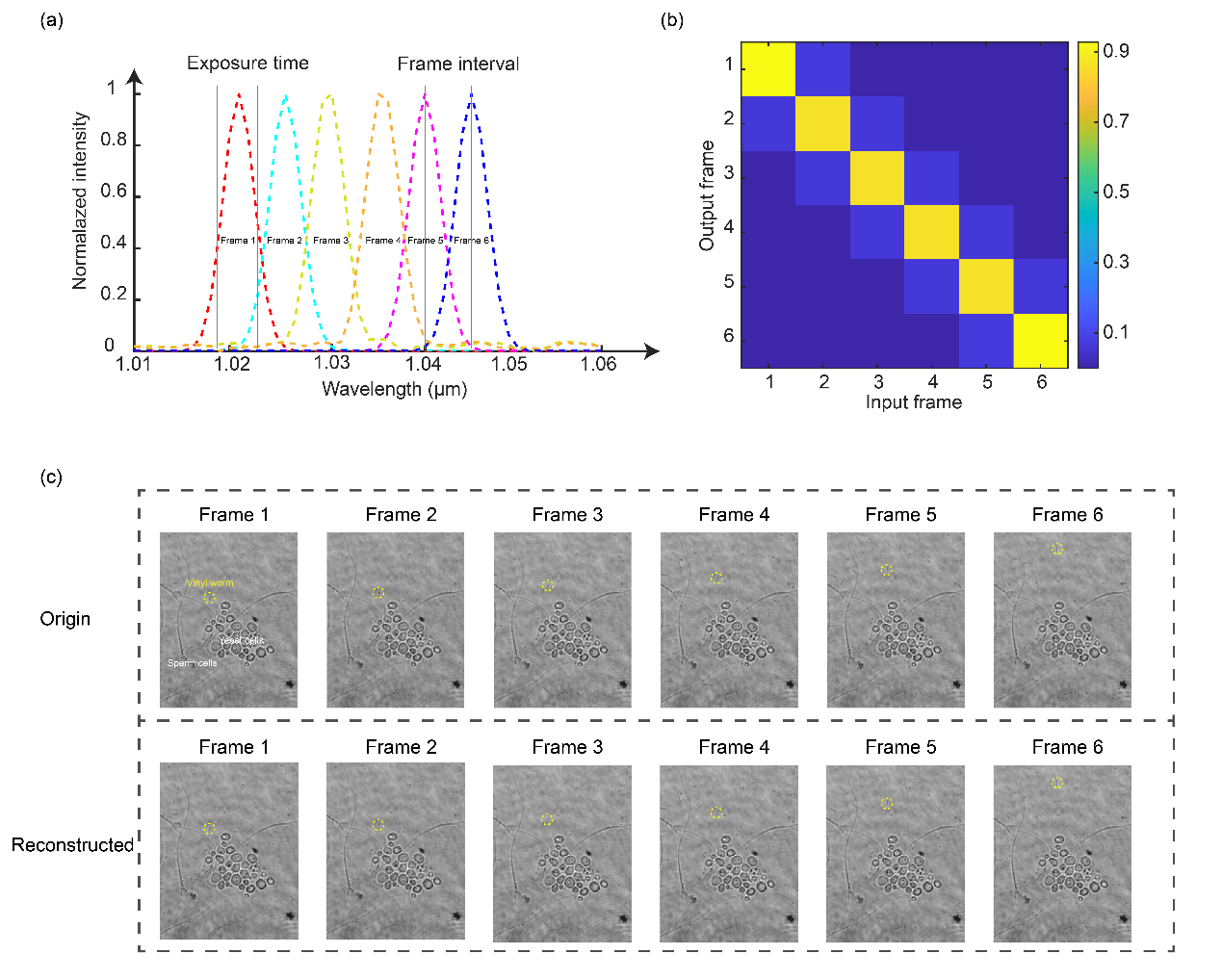


**Figure S11. Temporal mapping characteristics and cross-simulation imaging.** (a) Sliced-spectrum illustration with timing parameters. Normalized envelopes of spectral slices with exposure time (FWHM) and center-to-center frame interval indicated. (b) Inter-frame mixing matrix. Strongly diagonal with small nearest-neighbor terms, indicating 8% designed overlap. (c) Cross-simulation validation. Top: six original microscopic frames (yellow circles mark the trajectory of a motile organism); bottom: simulation-based reconstructions showing negligible artifacts.

**13. Laser-induced breakdown spectroscopy**

Laser-induced plasma emission spectroscopy (commonly referred to as LIBS) was conducted on Au and Ag targets under the same pump conditions used in the damage/plasma imaging experiments. The purpose of this measurement was not elemental identification, but to quantify the spectral content of parasitic self-emission generated during laser-metal interaction, which could otherwise contaminate the recorded BFP images. As shown in Fig. S12, the dominant emission from both Au and Ag is concentrated in the UV–visible range (Au: 270–320 nm; Ag: 280–330 nm), with negligible intensity in the near-infrared probe band used by ASUP. Based on this result, a long-pass filter (cut-on wavelength > 950 nm) was inserted in the detection path to suppress UV–visible self-emission and to ensure that the measured BFP images originate from the NIR probe light only, thereby preventing spectral cross-talk in the reconstruction.


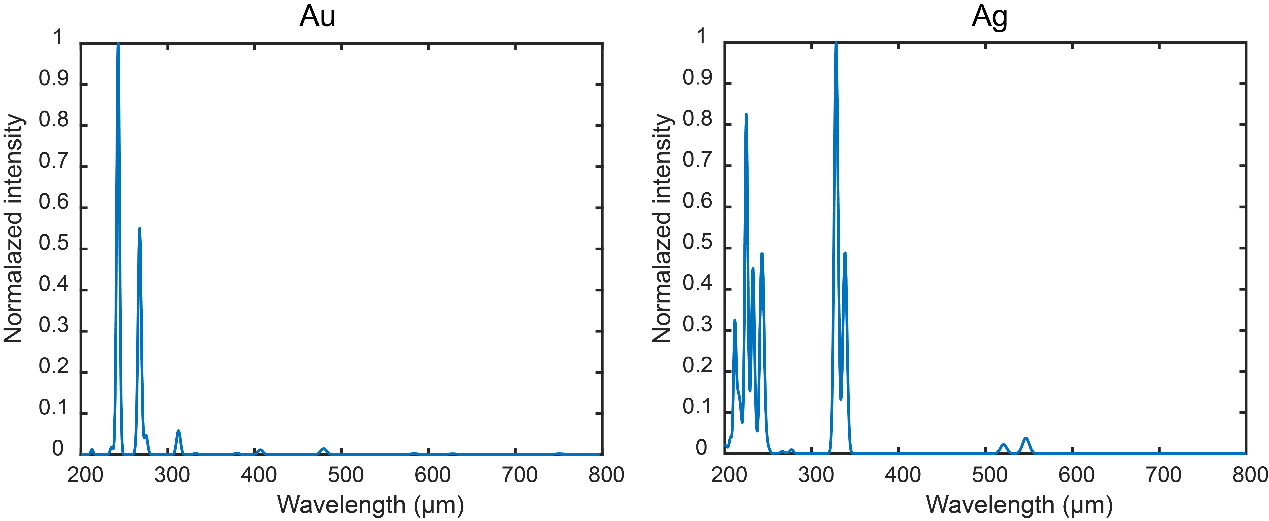


**Figure S12. Laser-induced breakdown spectra of Au and Ag.** Left and right panels show the normalized emission spectra of Au and Ag, respectively.

**14. Optical setup for transmission-type dynamic imaging**

To further illustrate the configuration of our ASUP-based system, a transmission-type imaging setup was employed to visualize the laser-induced plasma dynamics on a Ag thin film, as shown in Figure S13. A picosecond laser pulse irradiated an Ag-coated ground-glass substrate, generating a transient plasma plume. In this setup, the ultrafast laser beam was divided into a pump and a probe arm using a Brewster-angle polarizer and a half-wave plate. The pump beam was focused onto the sample to excite the plasma, while the probe beam passed through an optical delay line and illuminated the sample in transmission mode, enabling time-resolved shadowgraph imaging of the expanding plume. The transmitted probe was relayed through a series of objective lenses (OL1–OL3), a long-pass filter (LP), and MOC before being recorded by the detector. This configuration allowed ultrafast visualization of the plume expansion dynamics induced by laser irradiation.


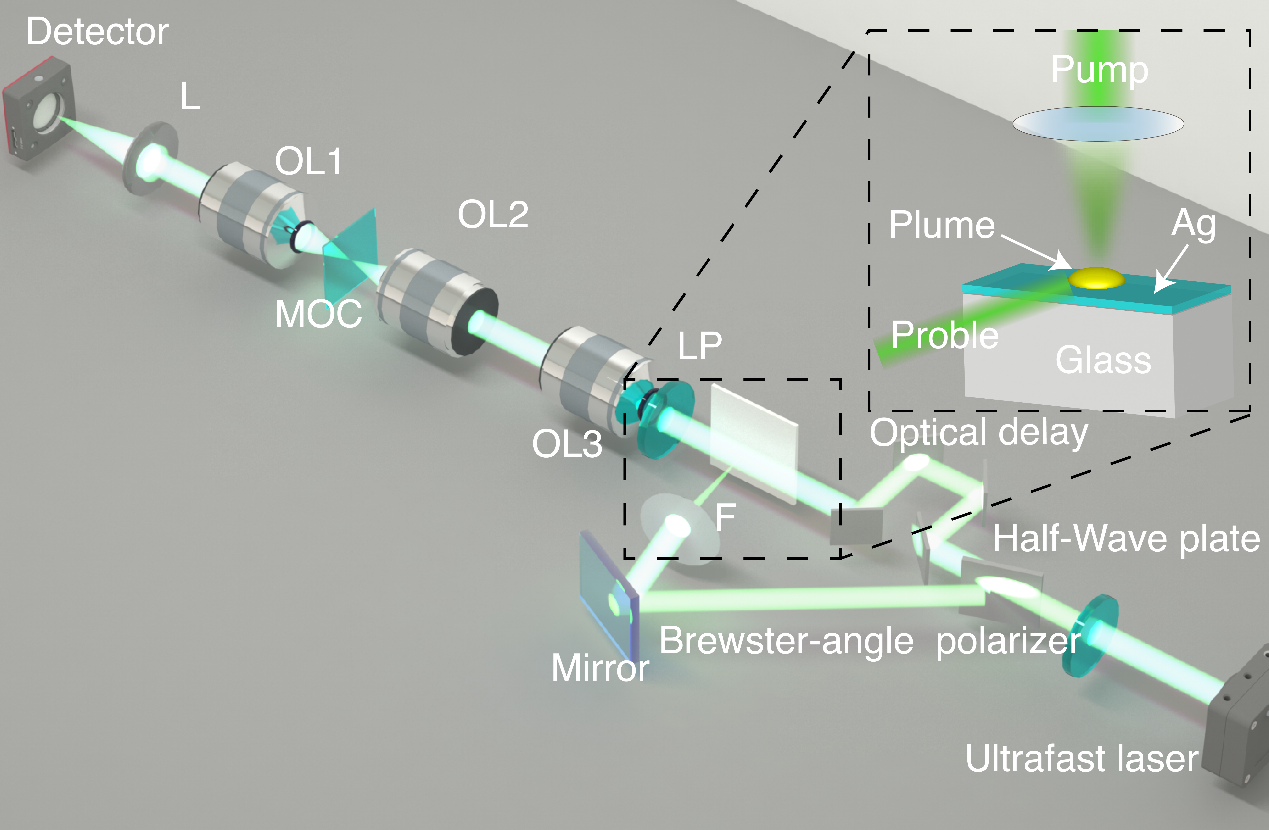


**Figure S13. Optical setup for transmission-type dynamic imaging in ASUP system.** The pump beam excites a plasma plume on the Ag film, while the time-delayed probe beam transmits through the sample to capture the ultrafast plume expansion using shadowgraph imaging.

**15. Physics-based angular-spectrum encoding validation and linear spectral reconstruction**

This section provides an explicit, physics-based forward model and a neural-network-independent linear inversion route for decoding spectra from BFP measurements. The goal is to demonstrate that the angular-spectrum encoding imposed by the MOC can be (i) modeled and calibrated deterministically, and (ii) used for direct spectral reconstruction via regularized least-squares inversion, thereby offering an interpretable baseline for comparison with the ERCNN decoder.

For a convergent beam, each BFP coordinate corresponds to a plane wave component characterized by an incident polar angle $\theta$ and azimuth angle $\phi$, i.e., a transverse wavevector $\mathbf{k}_{\perp}=(k_{x},k_{y})$. The MOC acts as a dispersive angular filter whose polarization-resolved transmission can be written in the s and p polarization as $\mathbf{T}(k_{x},k_{y},\lambda)=\left[ \begin{matrix} t_{s}(k_{x},k_{y},\lambda) & 0 \\ 0 & t_{p}(k_{x},k_{y},\lambda) \end{matrix} \right].$

For a given input polarization $\mathbf{e}_{in}$ and output polarization selection $\mathbf{e}_{out}$, the effective scalar OTF is (as derived in Supplementary Information Note 1)

$t(k_{x},k_{y},\lambda)=\mathbf{e}_{out}^{\dagger}\text{ }\mathbf{M}^{-1}\mathbf{T}(k_{x},k_{y},\lambda)\mathbf{M}\text{ }\mathbf{e}_{in}$ (S15.1)

where $\mathbf{M}$ is the basis-transform matrix between the (x, y) and (s, p) bases (see Eq.S1.2–S1.4).

Under general linear polarization, the transmitted intensity at each $\theta$ and $\phi$ channel can be expressed as a deterministic combination of $s$- and $p$-responses. Following a standard thin-film polarization treatment for a convergent beam, the total transmission intensity under a linear polarization angle $\psi$ can be written as

$I_{t}(\theta,\phi,\lambda;\psi)=A^{2}(\cos^{2}(\phi-\psi)\text{ }\mid t_{p}(\theta,\phi,\lambda)\mid^{2}+\sin^{2}(\phi-\psi)\text{ }\mid t_{s}(\theta,\phi,\lambda)\mid^{2})$ (S15.2)

where $A$ is the incident field amplitude in each angular channel. In our experiments (p-polarized incidence and no restrictive analyzer at detection), Eq.S15.2 reduces to the specific scalar form used in Supplementary Information Note 1 (Eq.S1.4), and the encoded BFP intensity map is

$I(k_{x},k_{y},\lambda)\propto\mid t(k_{x},k_{y},\lambda)\mid^{2}\text{ }\mid A_{in}(k_{x},k_{y},\lambda)\mid^{2}$ (S15.3)

When the illumination angular spectrum is fixed (or calibrated), the wavelength dependence of $I$ is governed primarily by the MOC transfer function, enabling a deterministic mapping from $\lambda$ to the BFP intensity pattern (as visualized in Fig. 2c).

To connect the physical encoding to an explicit inversion model, we discretize the BFP into $N$ measurement channels (pixels or binned $k$-space cells) indexed by $i=1,2,\ldots,N$. For each channel $i$, we define its spectral response function as

$g_{i}(\lambda)\text{ }\triangleq\text{ }I_{i}(\lambda)$ (S15.4)

Where $I_{i}(\lambda)$ is BFP intensity of channel $i$ under monochromatic input at λ. These response functions can be obtained either from the calibrated forward model in Eq.S15.1–S15.3 or directly from experimental wavelength scanning (as in Fig. 2d, where each $k$-space channel exhibits a distinct spectral dependence).

For an unknown incident spectrum $s(\lambda)$, the measured signal at the $i$-th angular channel can be expressed as a wavelength-weighted integral:

$y_{i}=\int_{\lambda_{\min}}^{\lambda_{\max}} g_{i}(\lambda)\text{ }s(\lambda)\text{ }d\lambda$ (S15.5)

where $y_{i}$ denotes the experimentally recorded value at channel $i$, $N$ is the number of angular channels used for reconstructio. Eq.S15.5 explicitly shows that the encoding process is deterministic and fully described by the calibrated functions $g_{i}(\lambda)$.

To reconstruct the unknown spectrum, the wavelength range of interest is discretized into $M$ spectral bins $\left\{ \lambda_{j} \right\}$. Eq.S15.5 can then be written in discrete form as

$y_{i}=\sum_{j=1}^{M} g_{i}(\lambda_{j})\text{ }s_{j}$ (S15.6)

where $s_{j}$ denotes the spectral intensity at $\lambda_{j}$. Collecting all channels yields a linear system

$y=\mathrm{Gs}$ (S15.7)

in which $G$ is the system matrix constructed from the calibrated angular-spectral responses $g_{i}(\lambda_{j})$. A straightforward NN-independent reconstruction can be obtained by solving a minimum-residual problem. In the absence of noise, this corresponds to a pseudoinverse solution. In practice, to improve robustness, we employ a regularized least-squares formulation:

$s^{*}=\arg\min_{s}{\parallel\mathrm{Gs}-y\parallel}_{2}^{2}+\alpha^{2}{\parallel s\parallel}_{2}^{2}$ (S15.8)

where $\alpha$ is a regularization parameter controlling the trade-off between reconstruction fidelity and noise amplification. The closed-form solution is

$s^{*}=\left( G^{\mathsf{T}}G + \alpha^{2}I \right)^{-1}G^{\mathsf{T}}y$ (S15.9)

Eq.S15.4–S15.9 demonstrate that, once the angular-channel response functions $g_{i}(\lambda)$ are calibrated, the mapping from BFP measurements to the incident spectrum is fully specified by a linear forward model and can be inverted without invoking a neural network. This confirms that the physical interpretability of the proposed system resides in the optical encoding stage provided by the MOC. The ERCNN employed in the main text does not define the measurement model, but instead serves as a data-driven numerical inversion tool that approximates and stabilizes the inverse mapping.

To further compare the performance of the two reconstruction strategies, we evaluated both the NN-independent linear inversion and the ERCNN-based decoding using the same encoded BFP measurements. Representative reconstruction results are shown in Figure S14, where the ground-truth spectra are compared with the reconstructed spectra obtained by the two methods.

As shown in Figure S14a, the NN-independent reconstruction based on regularized least-squares inversion is able to recover the main spectral features with good fidelity, yielding a coefficient of determination $R^{2}=0.928$ and SSIM of 0.904. This result confirms that the angular-spectrum encoding imposed by the MOC is sufficiently informative and that the underlying forward model is physically valid and invertible without relying on a neural network. In comparison, the ERCNN-based reconstruction (Figure S14b) achieves higher accuracy, with $R^{2}=0.987$and SSIM = 0.962. The learning-based decoder more effectively suppresses noise and residual cross-talk between neighboring spectral channels, and exhibits improved robustness against measurement imperfections and model mismatch. These advantages become increasingly important in practical experimental conditions, where finite signal-to-noise ratio and device nonidealities are unavoidable.

Therefore, in the present work, the NN-independent linear inversion serves as a transparent and physically interpretable baseline for validating the deterministic optical encoding, while the ERCNN is adopted as the primary decoding tool to achieve higher reconstruction fidelity and robustness in real experiments.


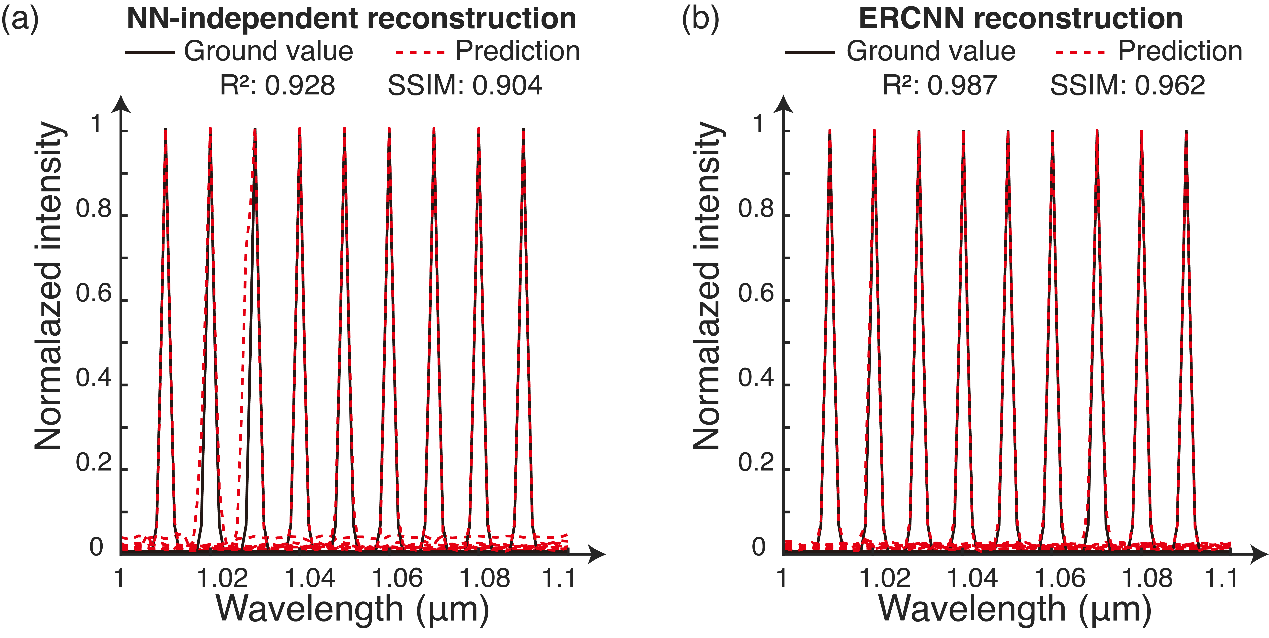


**Figure S14.** (a) Spectral reconstructions with NN-independent reconstruction (b) Spectral reconstructions with ERCNN reconstruction.

**16. Experimental demonstration of ASUP for ultrafast semiconductor dynamics and biological imaging**

In addition to the laser-metal interaction experiments presented in the main text, we experimentally demonstrate the applicability of ASUP to other classes of dynamic systems, including ultrafast carrier dynamics in semiconductors and spectrally resolved biological imaging. We first investigated laser-induced ultrafast reflectivity changes in germanium (Ge) and silicon (Si), which are governed by transient carrier excitation. In these experiments, a single 6 ps pump pulse with variable pulse energies (20–120 μJ) was used to excite the semiconductor surface, while a time-delayed probe pulse recorded the relative reflectivity change ΔR/R₀ at multiple delay times. Figure S15 summarizes the measured ultrafast reflectivity dynamics for Ge and Si. For both materials, a transient ΔR/R₀ is observed shortly after excitation, with the amplitude increasing monotonically with pump energy. This behavior originates from the rapid generation of high-density free carriers, which modifies the complex permittivity, leading to a transient reduction of the relative reflectivity. The subsequent recovery reflects carrier relaxation and recombination processes occurring on the picosecond timescale.


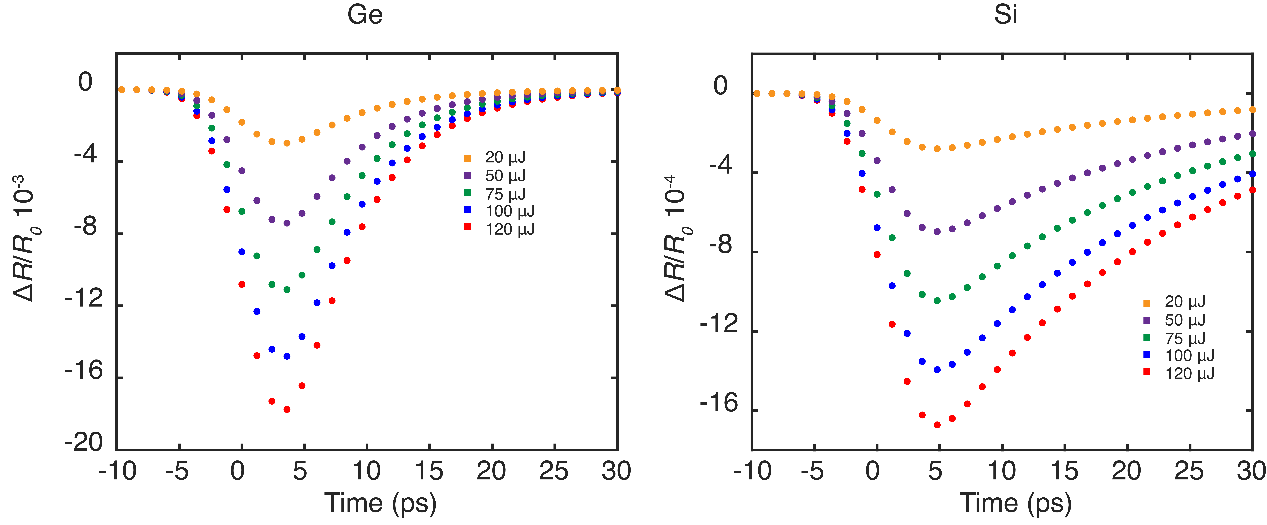


**Figure S15**. **Ultrafast transient reflectivity dynamics of semiconductors.** Time-resolved relative reflectivity change (ΔR/R₀) of (a) Ge and (b) Si under single pulse 6 ps laser excitation with different pulse energies (20, 50, 75, 100, and 120 μJ).

To further illustrate the capability of ASUP in biological contexts, we performed multispectral imaging of various biological specimens using the distinct spectral frames inherently encoded by the ASUP system. Instead of relying on repetitive wavelength scanning, each ultrafast frame corresponds to a specific spectral band of the chirped probe pulse. Figure S16 presents representative reconstructed images of different biological samples, including Marchantia polymorpha cyphella, antheridia of Polytrichum commune, pear stone, tomato flesh, Lilium ovary, fern stem, onion root undergoing mitosis, and dicotyledon stem tissue, acquired at center wavelengths ranging from 1020 nm to 1045 nm. Together, these demonstrations highlight the versatility of ASUP as a single-shot ultrafast imaging platform, capable of probing a broad range of physical and biological systems with inherent spectral discrimination.


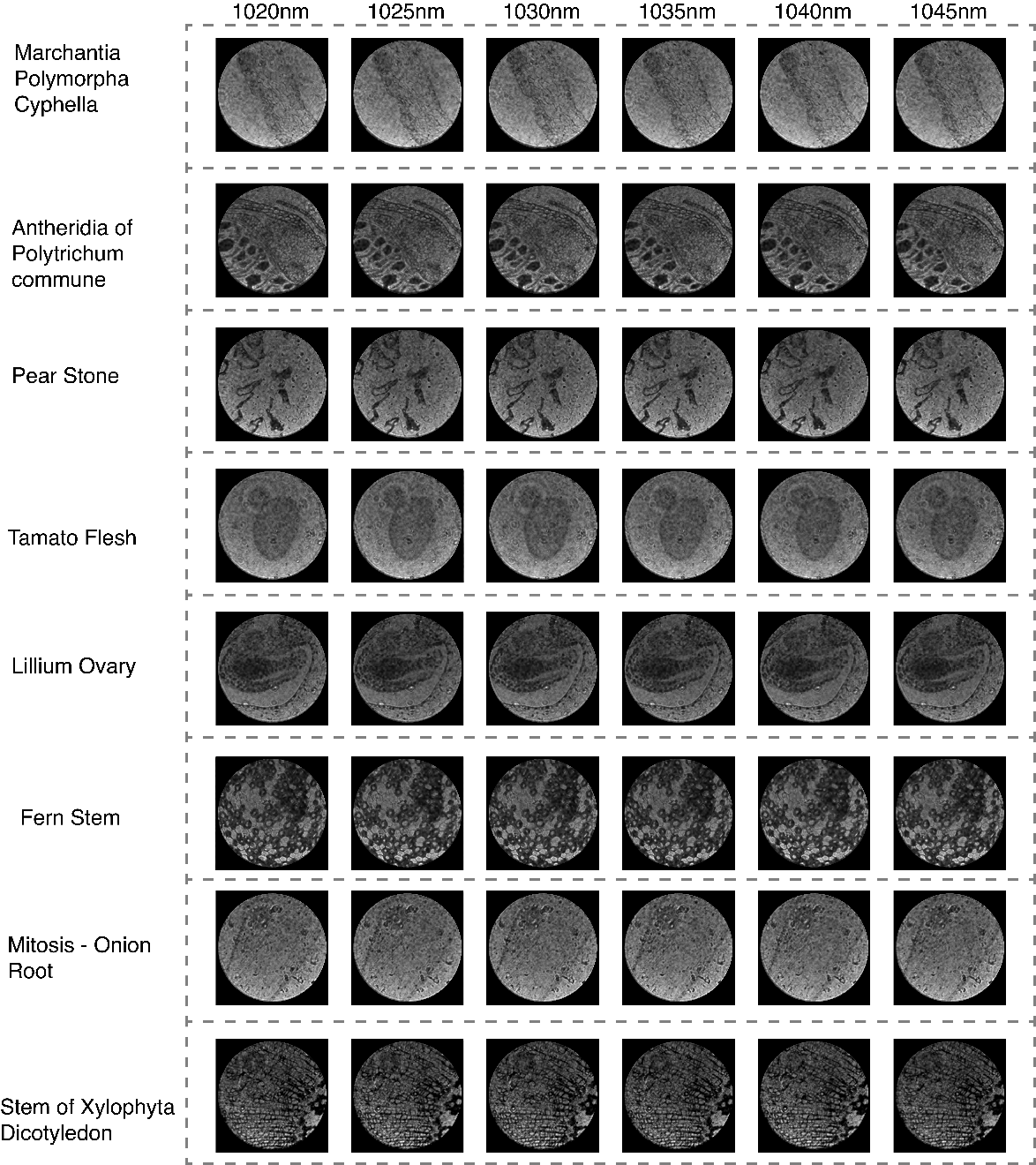


**Figure S16. Spectrally resolved biological imaging using ASUP**. Reconstructed images of various biological specimens at different spectral frames corresponding to center wavelengths of 1020, 1025, 1030, 1035, 1040, and 1045 nm.

**17. Definition of the effective temporal encoding window**

This note clarifies the definition of the temporal window used for ultrafast frame encoding. The wavelength-time mapping of the chirped probe pulse was experimentally characterized using FROG, revealing a linear relationship within the central spectral region. Only this spectrally selected working band, where both the signal intensity and the chirp linearity are maximal, is used for frame reconstruction. This region corresponds to an effective temporal window of approximately 6 ps, which defines the total encoded duration reported in the main text. Spectral components outside this window are excluded and do not contribute to frame formation.


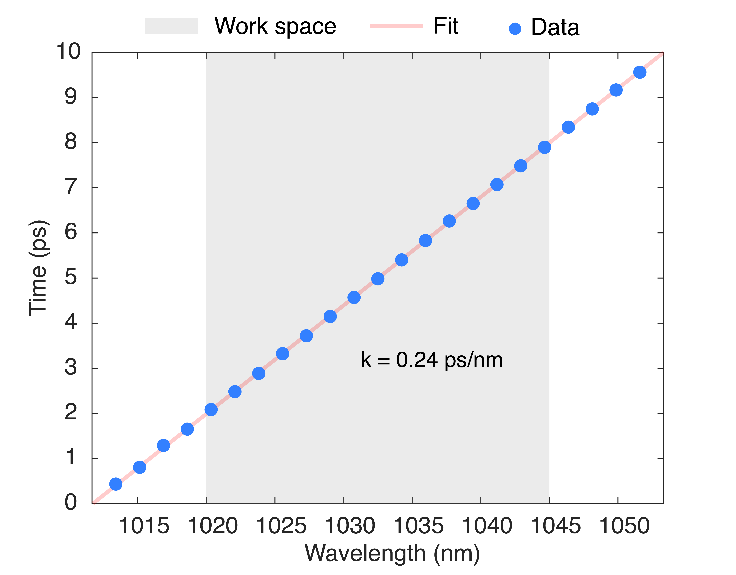


Figure S17. Wavelength-time mapping and effective temporal encoding window.

**Reference:**

1. Joannopoulos, J. D. et al. *Photonic Crystals: Molding the Flow of Light*. 2nd ed. Princeton University Press (2011).

2. Verslegers, L. et al. Temporal coupled-mode theory for resonant apertures. *Journal of the Optical Society of America B* 27, 1947-1956 (2010).

3. Long, O. Y. et al. Isotropic topological second-order spatial differentiator operating in transmission mode. *Optics Letters* 46, 3247 (2021).

4. Deng, W. et al. Electrically tunable two-dimensional heterojunctions for miniaturized near-infrared spectrometers. *Nature Communications* 13, 4627 (2022).

5. Yoon, H. H. et al. Miniaturized spectrometers with a tunable van der Waals junction. *Science* 378, 296-299 (2022).

6. Yuan, S. et al. A wavelength-scale black phosphorus spectrometer. *Nature Photonics* 15, 601-607 (2021).

7. Fan, Y. et al. Dispersion-assisted high-dimensional photodetector. *Nature* 630, 77-83 (2024).

8. Yako, M. et al. Video-rate hyperspectral camera based on a CMOS-compatible random array of Fabry-Pérot filters. *Nature Photonics* 17, 218-223 (2023).

9. Zhou, Z. et al. Electrically tunable planar liquid-crystal singlets for simultaneous spectrometry and imaging. *Light: Science & Applications* 13, (2024).

10. Nakagawa, K. et al. Sequentially timed all-optical mapping photography (STAMP). *Nature Photonics* 8, 695-700 (2014).

11. Dong, J. et al. Single-shot ultrafast terahertz photography. *Nature Communications* 14, 1704 (2023).

12. Ding, P. et al. Single-shot polarization-resolved ultrafast mapping photography. *Science Bulletin* 68, 473-476 (2023).

13. Tang, H. et al. Single-shot compressed optical field topography. *Light: Science & Applications* 11, 244 (2022).

14. Yao, Y. et al. Single-shot real-time ultrafast imaging of femtosecond laser fabrication. *ACS Photonics* 8, 738-744 (2021).

15. Zeng, X. et al. High-spatial-resolution ultrafast framing imaging at 15 trillion frames per second by optical parametric amplification. *Advanced Photonics* 2, (2020).

16. Li, Z. et al. Single-shot tomographic movies of evolving light-velocity objects. *Nature Communications* 5, 3085 (2014).

17. Yan, L. et al. Multi-frame observation of a single femtosecond laser pulse propagation using an echelon and optical polarigraphy technique. *IEEE Photonics Technology Letters* 25, 1879-1881 (2013).

18. Kakue, T. et al. Digital light-in-flight recording by holography by use of a femtosecond pulsed laser. *IEEE Journal of Selected Topics in Quantum Electronics* 18, 479-485 (2012).

19. Ehn, A. et al. FRAME: femtosecond videography for atomic and molecular dynamics. *Light: Science & Applications* 6, e17045 (2017).

20. Moon, J. et al. Single-shot imaging of microscopic dynamic scenes at 5 THz frame rates by time and spatial frequency multiplexing. *Optics Express* 28, 4463-4474 (2020).

21. Zhu, Q. et al. FISI: frequency domain integration sequential imaging at 1.26×10^13 frames per second and 108 lines per millimeter. *Optics Express* 30, 27429 (2022).

22. Suzuki, T. et al. Single-shot 25-frame burst imaging of ultrafast phase transition of Ge2Sb2Te5 with a sub-picosecond resolution. *Applied Physics Express* 10, 092502 (2017).

23. Suzuki, T. et al. Sequentially timed all-optical mapping photography (STAMP) utilizing spectral filtering. *Optics Express* 23, 30512 (2015).

24. Zhu, Y. et al. All-optical high spatial-temporal resolution photography with raster principle at 2 trillion frames per second. *Optics Express* 29, 27298 (2021).

25. Lu, Y. et al. Compressed ultrafast spectral-temporal photography. *Physical Review Letters* 122, 193904 (2019).

26. Meng, Y. et al. High-channel spectral-temporal active recording (H-STAR) for femtosecond scenes observation in a single-shot. *ACS Photonics* 11, 419-427 (2024).

27. Liu, J. et al. Swept coded aperture real-time femtophotography. *Nature Communications* 15, 1589 (2024).

28. Meng, Y. et al. High-frequency enhanced ultrafast compressed active photography. *Opto-Electronic Advances* 8, 240180-240180 (2025).

29. Gao, L. et al. Single-shot compressed ultrafast photography at one hundred billion frames per second. *Nature* 516, 74-77 (2014).

30. Mishra, Y. N. et al. Single-pulse ultrafast real-time simultaneous planar imaging of femtosecond laser-nanoparticle dynamics in flames. *Light: Science & Applications* 13, 221 (2024).

31. Liang, J. et al. Single-shot stereo-polarimetric compressed ultrafast photography for light-speed observation of high-dimensional optical transients with picosecond resolution. *Nature Communications* 11, 5252 (2020).

32. Lai, Y. et al. Single-shot ultraviolet compressed ultrafast photography. *Laser & Photonics Reviews* 14, 2000122 (2020).

33. Yang, C. et al. Hyperspectrally compressed ultrafast photography. *Physical Review Letters* 124, 023902 (2020).

34. Liang, J. et al. Single-shot real-time femtosecond imaging of temporal focusing. *Light: Science & Applications* 7, 42 (2018).

35. Liang, J. et al. Single-shot real-time video recording of a photonic Mach cone induced by a scattered light pulse. *Science Advances* 3, e1601814 (2017).

36. Feng, X. H. & Gao, L. Ultrafast light field tomography for snapshot transient and non-line-of-sight imaging. *Nature Communications* 12, 2179 (2021).

37. Lin, Z. et al. Electron-phonon coupling and electron heat capacity of metals under conditions of strong electron-phonon nonequilibrium. *Physical Review B* 77, 075133 (2008).

38. Tsibidis, G. D. et al. Damage threshold evaluation of thin metallic films exposed to femtosecond laser pulses: The role of material thickness. *Optics and Laser Technology* 156, 108484 (2022).

39. Chen, A. M. et al. Modeling of femtosecond laser damage threshold on the two-layer metal films. *Applied Surface Science* 257, 1678-1683 (2010).

40. Chen, A. et al. Ultrafast investigation of electron dynamics in the gold-coated two-layer metal films. *Thin Solid Films* 529, 209-216 (2013).
